# Habitat suitability and the genetic structure of human populations during the Last Glacial Maximum (LGM) in Western Europe

**DOI:** 10.1371/journal.pone.0217996

**Published:** 2019-06-19

**Authors:** Colin D. Wren, Ariane Burke

**Affiliations:** 1 Department of Anthropology, University of Colorado Colorado Springs, Colorado Springs, Colorado, United States of America; 2 Département d'Anthropologie, Université de Montréal, Montreal, Québec, Canada; Max Planck Institute for the Science of Human History, GERMANY

## Abstract

Human populations in Western Europe during the Last Glacial Maximum were geographically constrained to glacial refugia by the severity of the climate and ecological risk factors. In this research we use an agent-based model of human mobility and interaction, based on ethnographic and archaeological data, to explore the impact of ecological risk on human population structure via a reconstructed landscape of habitat suitability. The agent-based model allows us to evaluate the size and location of glacial refugia, the size of the populations occupying them and the degree of genetic relatedness between people occupying these areas. To do this, we model the probability of an agent foraging groups’ survival as a function of habitat suitability. The model’s simulated “genomes” (composed of regionally specific genetic markers) allow us to track long-term trends of inter-regional interaction and mobility. The results agree with previous archaeological studies situating a large glacial refugium spanning southern France and northeastern Spain, but we expand on those studies by demonstrating that higher rates of population growth in this central refugium led to continuous out-migration and therefore genetic homogeneity across Western Europe, with the possible exception of the Italian peninsula. These results concur with material culture data from known archaeological sites dating to the Last Glacial Maximum and make predictions for future ancient DNA studies.

## Introduction

This paper explores the impact of environmental constraints on the size, distribution and structure of human populations living in Western Europe during the Last Glacial Maximum 19–23 kyrs BP). A habitat suitability (HS) index developed elsewhere [1] is used to establish the characteristics of a landscape that affects the spatial distribution and probability of survival of regional populations. We then use an agent-based model to explore the impact of different scales of mobility on the size and level of connectivity between regional populations, as well as their genetic makeup.

Since the demographic structure of a population has repercussions for cultural evolution, affecting rates of cultural innovation for example [2–4], this research has implications for the evolution of the biological and cultural landscape of Western Europe at the height of the last Glacial.

### Timeframe of interest

The Last Glacial period (encompassing Marine isotope stages 4–2) is characterised by a global trend towards cooler and drier conditions, culminating with the Last Glacial Maximum (LGM), which correlates with Greenland Stadial 2.1 and marks the maximum volume of global ice and consequently, the lowest sea-stand [5–7]. Proxy-based climate data indicate that relatively cold and dry conditions prevailed in Europe at this time, accompanied by a decline in arboreal pollen and the expansion of the Eurasian steppe [8,9]. Various paleoclimate models produce comparable results [10–12]. The downward trend in temperature and precipitation rates that characterises the Last Glacial was punctuated by a series of abrupt, millenial-scale climate shifts, well-documented in the Greenland ice-core records [8,9]. These climate shifts may have played a role in the timing of modern human dispersals into Europe [13]. The onset of extreme conditions during the LGM is thought to have patterned the subsequent distribution of human populations within Europe [14–16].

**Fig 1:**
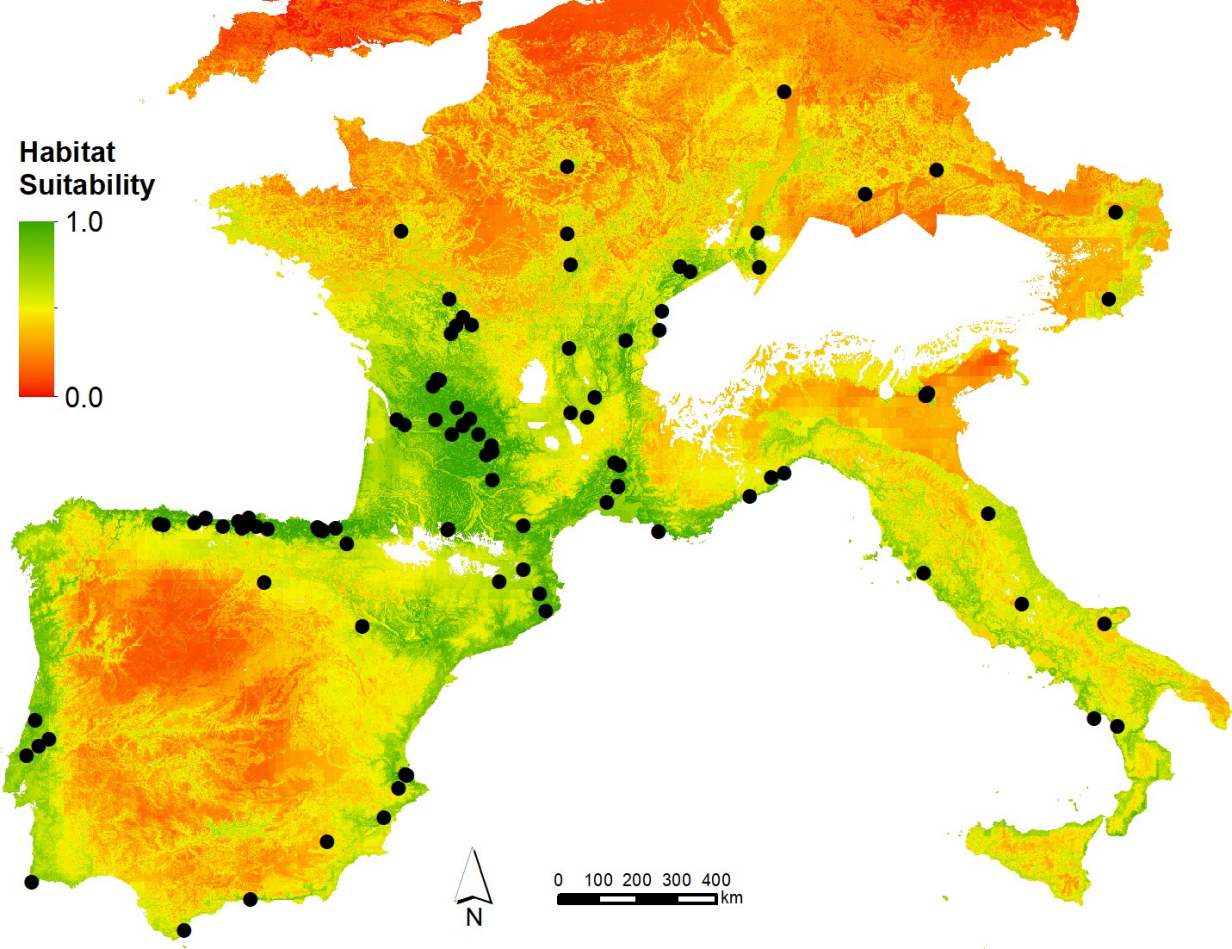
Spatial distribution of habitat suitability values derived in Burke et al. (2017). Higher values of habitat suitability indicate cells that are more favourable for human habitation. Points represent known LGM period archaeological sites. Note that known glacial extents are masked. European border polygons republished from GADM.org under a CC BY license, with permission from GADM, original copyright 2018.

The archaeological record indicates that human populations withdrew from Northern Europe and other previously inhabited regions, such as the centre of the Iberian Peninsula, during the LGM (Fig 1). Whether the populations living in these regions crashed or whether they migrated to glacial refugia is not currently known, but it seems likely that overall population numbers fell during this time interval [17]. Paradoxically, the record also shows that the LGM was a period of cultural innovation, marked by the regional fragmentation of material culture [18–20]. The advance of the ice-sheets and the retreat of human populations to southern, glacial refugia during the LGM hypothetically created a situation where smaller, regional populations became spatially isolated, producing new and divergent cultural patterns. This research allows us to address the question of the regionalisation of material culture in Western Europe during the LGM by modelling human population dynamics, including individual mobility, inter-regional connectivity and regional survival rates as a function of the structure of the environment, represented by a model of habitat suitability which acts as a proxy.

### Habitat suitability models

Underpinning this research is a model of habitat suitability for the LGM derived from the application of a machine-learning algorithm (Random Forest) to the spatial distribution of prehistoric sites and a suite of geographical and paleo-environmental variables [1]. Archaeologists use models such as this both predictively, e.g. to design archaeological surveys, and interpretively, as a means of identifying key predictors of human spatial behaviour. Climate variables such as precipitation rates and temperature are usually included in the modelling process because they regulate vegetation cover, which in turn determines net primary productivity (NPP), or available biomass. Geographical variables such as latitude, continentality, and elevation are included because they mitigate climate conditions and produce spatial and temporal variation in the distribution of resources key to human survival.

The advent of coupled global climate models combining atmospheric and oceanic circulation (GCMs) greatly enhanced our ability to produce accurate climate simulations of past and present conditions. The Paleoclimate Model Intercomparison Project (PMIP) was designed to test the ability of different GCMs to simulate climate under conditions of rapid or extreme climate change, focussing initially on the Last Glacial Maximum (centered at 21ka) and the mid-Holocene [21]. These paleoenvironmental simulations allowed scientists to compare and evaluate the relative performance of different model architectures, independently validating results using climate proxies derived from terrestrial and marine sediment records [16,21]. The IPSL-CM5 model used in this research compares very favourably with other GCMs participating in PMIP [12].

GCMs produce outputs on fine temporal but coarse spatial scales. Two methods exist for downscaling GCM outputs to a more appropriate scale for archaeological research: the use of nested regional dynamic models or statistical downscaling methods. The Stage 3 project, for example, which marks a watershed in the development of multidisciplinary research into human-environment interactions, used a conventional GCM coupled with a regional circulation model for Europe (REGCM2) to produce a meso-scale analysis (~60 km x 60 km) exploring the impact of climate change on Neanderthal and human populations during the last Glacial period [22,23]. The second approach, which is the one adopted by the Hominid Dispersal Research Group (HDRG), uses a statistical method to downscale the GCM outputs as a function of topography, continentality and temperature [24–26] producing simulated climate conditions at a fine spatial scale (15x15 km^2^). This approximates the scale of the “catchment” areas around archaeological sites, i.e., the area within which human activities took place on a daily basis, as suggested by Vita-Finzi and Higgs [27].

GCMs have been used elsewhere to produce spatial models of human spatial distribution during the LGM [28–33]. The various archaeological models that have been produced to date are broadly similar, which is understandable since they are based on essentially the same archaeological dataset (or subsets thereof) and comparable suites of candidate predictors. Predictors typically include elevation, slope, maximum and minimum temperatures and maximum and minimum precipitation rates. The devil is in the detail, however, and the habitat suitability model used in this research differs fundamentally from other models in its explicit use of climate variability (defined as deviation over a defined time-frame compared with long-term statistics) as a potential predictor of habitat suitability. Thus defined, climate variability is a measure of how predictable conditions are from year to year (the timeframe defined in our original study) with repercussions for the distribution of plant and animal resources. Numerical climate models are generally considered to underestimate interannual variability [34], at least under modern conditions, but still provide plausible physical scenarios with which to test the impact of variability on habitat suitability.

Hunter-gatherers use mobility as a key strategy for dealing with changes in the distribution of resources. Ethnographically known hunting and gathering groups maintain a fluid social structure and extended social networks that promote the gathering of information about resource availability and facilitate residential moves in the event of resource failure. Mobility has inherent costs that need to be balanced out against expected returns, however, and the structure of the environment, i.e., the pattern of distribution of both resources and resource risk, affects the degree of mobility in human systems [35,36]. Consequently, the HDRG team included several indices of climate variability in the model-building process in addition to a suite of conventional climate and topographic variables. The model-building methodology is described in full elsewhere [1]. In initial research at the scale of the Iberian Peninsula [16] and subsequent research on a larger European domain [1] climate variability consistently emerged as one of the predictors of archaeological site location. The habitat suitability model with the best performance (run 26) resulting from the 2017 publication is the one that is used in this research. According to this model, habitat suitability (HS) is determined by seven predictors: elevation, slope, variability in Spring precipitation rates, maximum monthly average temperature in Autumn, variability in monthly temperature calculated using the Standard Temperature Index, standard deviation in average monthly temperature in Winter, and the standard deviation in average monthly temperature in Autumn. Our working hypothesis is that Palaeolithic populations will have attempted to minimise ecological risk by avoiding regions where HS is relatively low.

## Methods

We created an ABM (LGM_ecodynamics 1.1) that is a modification of hominin_ecodynamics 2.0 designed by Barton and Riel-Salvatore [37]. The purpose of LGM_ecodynamics (download here: https://doi.org/10.25937/na38-tj46) is to evaluate to what extent the heterogeneous habitat of Western Europe during the LGM affected the structure and connectivity of the metapopulation (a regional grouping of connected populations of a species [38]) and what effect that may have had on the distribution and survival of regional populations. We model the mobility of individual hunter-gatherer families (“agents”) within annual territories and the long-term population dynamics as agents interact with the landscape and with one another. Following the original model, agents maintain home-range fidelity by moving within a specified radius (*fradius*) around a fixed central home cell. Note that movement occurs by “teleportation”, i.e., intermediate cells are ignored and we do not explicitly model the relative differences in the cost of travel. However, the probability that a given cell will be selected for movement decreases with distance which, with a monthly time step, works as a loose proxy for cost of movement. Large-scale shifts in the spatial distribution of the metapopulation occur as newly produced agents are deposited on a new cell and are affected by differential mortality rates linked to habitat suitability (HS).

We experiment with several parameters, including: type of mobility, home range size, maximal distance for a given move, and a parameter that quantifies the impact of HS on survival rates. In each experiment, we investigate the effects of the parameters on the propagation of neutral genetic traits and on population size both at the regional and metapopulation levels. Finally, we ask whether the observed patterns of genetic exchange help explain the distinct material culture groupings reflected in the archaeological record.

Below we summarize only the most pertinent aspects of the model. The key differences that distinguish our model from Barton and Riel-Salvatore’s model are: 1) we model only modern humans rather than Neanderthals and modern humans, so all of the agents have the same behaviours; 2) we model multiple regional populations instead of just two species, and; 3) the landscape is heterogeneous and impacts movement. The full model description in the standardized ODD protocol format is included in the supplemental information (S1 Appendix).

### Regional designations

**Fig 2:**
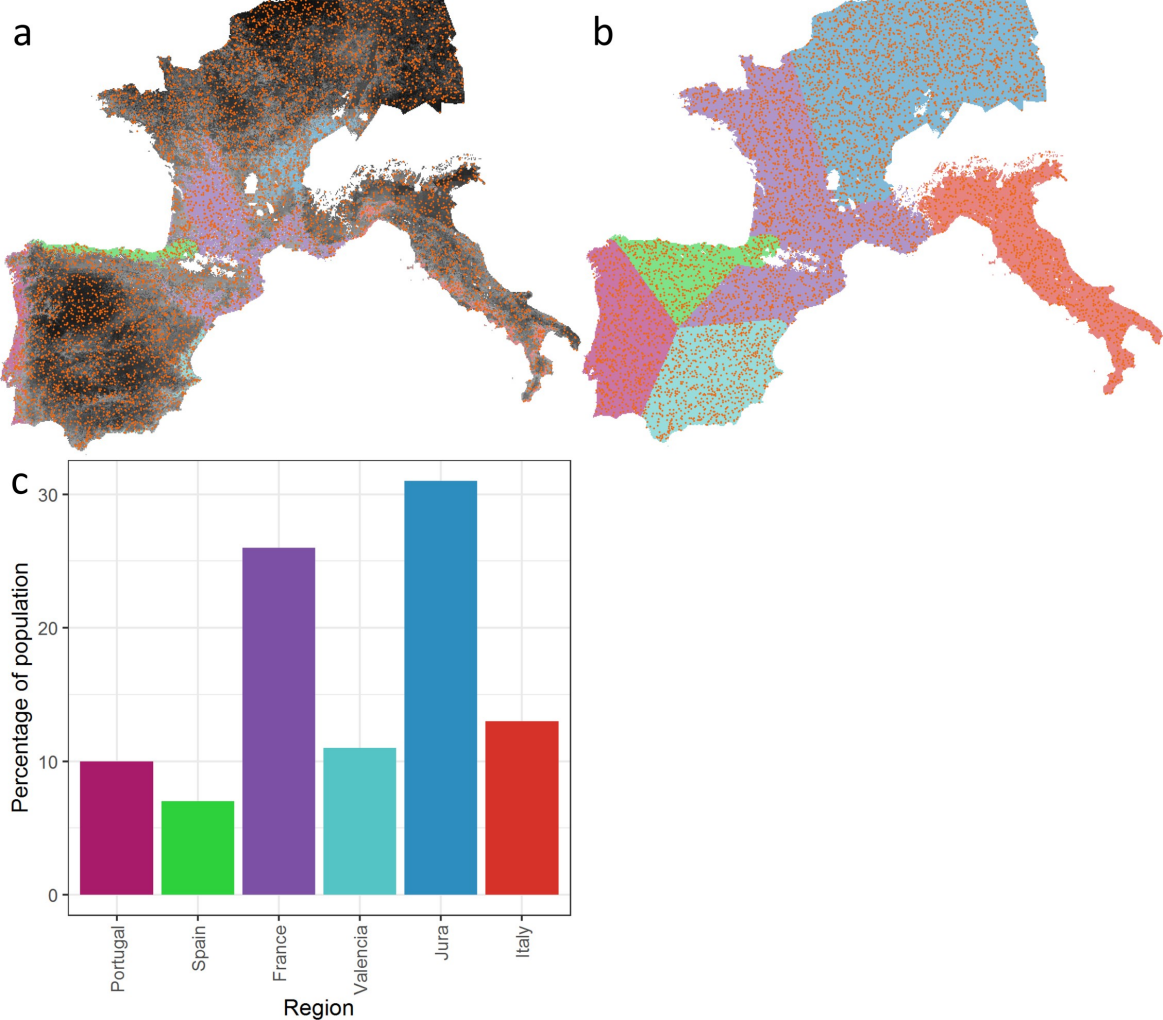
Example initial metapopulation of 7000 randomly distributed agents (coloured dots) overlaid on a) lighter coloured “core area” cells determined by the hotspot analysis, b) larger regions used for counting agent regional population sizes, and c) initial distribution of 7000 agents across the regions.

Rather than model a homogenous landscape, we import a heterogeneous landscape based on the predictive model of habitat suitability (HS) (see above). This suitability landscape forms the landscape of the ABM. Instead of imposing regional population designations from previously published literature (such as Solutrean or Epigravettian for example), we use a spatial analysis procedure of the habitat suitability landscape itself to derive “hot-spots” of spatially clustered high HS-value cells. Specifically, we used Hot Spot Analysis Getis-Ord Gi* tool in ArcGIS 10.5 [39] with the distance threshold computed by the tool and then used all cells binned as +1 or above. We grouped the resulting clusters based on contiguous cells (Moore neighbourhood) using ArcGIS’s Region Group tool, and further grouped small isolated clusters of cells into larger groupings by hand digitizing large polygons with the borders running along a midline between each pair of clusters to produce six distinct clusters which we numbered sequentially (Fig 2A, see S1 Appendix for additional details). These clusters form the core areas of our “regions” and are the basis for attributing a genetic fingerprint to the agents (see below). The six “regions” (Fig 2B) are used as a heuristic device to track agent movements during the experiments (see below). Note that the model does not use the core area or region values to influence agent movement but only to track the movement and reproduction patterns of the metapopulation. Each 5 by 5 km cell in the ABM, therefore, has three attributes: habitat suitability (0–1), core area (1 to 6), and region (1 to 6).

The six core areas have similar HS values but are unequal in size, with the core area centered in France being far larger than the others (Table 1). The six regions have quite different cell frequencies across the range of habitat suitability values.

**Table 1:** Summary data on the habitat suitability landscape per core area and region.

	Count of cells	Mean HS	StDev HS
**Core area**			
Portugal	1353	0.70	0.06
N Spain	1431	0.77	0.11
France	8266	0.76	0.11
Valencia	418	0.69	0.07
Jura	1772	0.69	0.09
Italy	992	0.69	0.07
**Region**			
Portugal	11953	0.38	0.18
N Spain	7887	0.42	0.23
France	30439	0.53	0.19
Valencia	13214	0.34	0.14
Jura	37357	0.29	0.17
Italy	16516	0.42	0.13

### Initial ABM model parameters

#### Populating the initial landscape

It is notoriously difficult to estimate the size of past populations. The historically uneven distribution of research effort, dating errors and differential patterns of site preservation due to regional taphonomic processes all contribute to biases in the archaeological record. Efforts to model the size of the metapopulation living in Europe during the LGM using different methods [31–33,40] result in widely divergent estimates of population size. Results range from an average of 5585 people [40] to a minimum of 130,000 people [33] for Europe, and 3,046,000–8,307,000 people for the Old World [31]. Differences in estimated population sizes are attributable to the use of different methods of estimating the total habitable area (e.g., using different climate predictors) and/or different growth rate estimates. Our experiments begin with an initial metapopulation of 7000 agents, though model dynamics (including growth rate) quickly affect the population size. Agents are abstract tokens, conceptually they can be considered to represent family groups of at least two adults and a variable number of dependents. If we assume an average family size of 4–5 people (following [41] p. 172), this figure falls within the estimated range provided for European populations during the LGM by Bocquet-Appel et al. (Table 5 in [40]).

Each model run begins with the agents randomly scattered across the landscape (Fig 2A and 2B). Each agent has a genome represented by a list of trait pairs that is *trait_list_size* in length, defaulting to 100. Each agent is initialized with a genome full of zeros. As initialized agents move and touch a cell with a core area for the first time, they acquire a genetic fingerprint, i.e., a genome of traits representing that core area’s code (e.g. 1 for Portugal, 2 for northern Spain, etc.). When two agents create a new agent (see next section for population dynamics) the new agent’s genome contains a randomly assorted combination of the two “parent” agents’ genomes. This procedure allows us to track the long-term inter-generational migration history of the regional populations (Box 1).

**Box 1. Agent genome**

Initialised agent: (0000000000000000000000000000000000000000000000000000…)

Agent touches a *core area* cell of Portugal: (1111111111111111111111111111111111111111111111111111…)

New agent created via a reproduction event with an agent from Jura *core area*:

(1441411144141141141444441114414114441114444114144414…)

The alleles within the agents’ genomes are composed of entirely neutral traits with respect to behaviour. The analogy to the biological concept of a genome refers to the randomized assortment of parental traits over generations. This is useful for representing the relative contribution of different regions’ traits to the metapopulation over the duration of a simulation run. For example, a small regional population’s traits will likely be lost or greatly diluted when mixing frequently with a much larger population [42] and will also likely be retained if relatively isolated from other groups due to effects of the landscape.

### Population dynamics

We model the probability of agents disbanding or persisting, and the probability of creating new agents using life tables drawn from Pennington [43] and population growth rates derived from the ethnographic and archaeological literature. Pennington’s life tables contain the basic parameters that govern population growth rates. We use the birthrate calculated from these tables as a proxy for the probability that an agent may generate a new household.

To calculate the birthrate, we use Pennington’s [43] summary of the total number of births over a woman’s lifespan, i.e. the total fertility rate (TFR), for a variety of hunter-gatherer groups. Among the hunter-gatherer populations sampled, the TFR ranges from 2.6 to 8.0 births with a median around 4.3 (Table 7.4 in [43]). The hunter-gatherer groups in this sample live in a variety of environmental settings but the early Kutchin are a good proxy for LGM populations in Western Europe since they lived a traditional hunting and gathering lifestyle in subarctic conditions in the Canadian Yukon; their TFR is 4.4 (Ross 1981 cited in [43]). Although estimates of the age of first birth and last birth vary widely, the reproductive span of hunter-gatherer women extends from adolescence to about 40 years of age [43] (p. 175), therefore we assume an average reproductive span of 25 years. We took the Kutchin TFR of 4.4 and divided it by 25 to derive an annual *birthrate* of 17.6%.

The model runs with a time step representing one month. Over the course of each run, therefore, agents generate new households at a specified probability equal to *birthrate* divided by 12. This new *birthrate* parameter is the probability that agents will found a new household with the nearest other agent. Our agents are exogamous and only realise their potential for producing new agents when there is an empty cell within their *fradius*

As a default, the *deathrate*, or probability of an agent household disbanding in each time step, is set to the same value as the *birthrate* which means the net population growth rate is zero. The LGM is generally thought to represent a period of initial population contraction, followed by a period of little or no population growth in Europe, so assuming zero growth in our initial model is an appropriate first step [40]. We then experiment with variable effects of the environment on the *deathrate* as explained below.

We used two initial experiments (see SI) to ensure that our model produces patterns consistent with the growth rates reported for ethnographically or archaeologically described hunter-gatherer populations, which is between 0 and 0.04% (e.g., [43–46]). We then test the impact of habitat suitability on agent deathrates which in turn affects the size and distribution of regional populations in Europe. This has the dual advantage of allowing us to explain the components of the final model piece by piece and carefully investigate the effects of each additional component on the model results.

#### Mobility

Agents move about the landscape at each time step within a given radius around their home cell (the home cell is randomly assigned at the beginning of the run). The *fradius* parameter sets the search radius used by agents when making decisions about where to move. Note that the home cell does not change during the life of the agent, i.e. agents are restricted to an annual territory, centered on their home cell, whose size is set by *fradius*. Agents move at each time step and may not move onto an already occupied cell, or a cell that was occupied by another agent during the previous time step, but their mobility radii (territories) may overlap. Although cost of movement is not quantified, agent movement is a weighted random-walk with cells at greater distances being weighted lower, thereby making shorter movements preferred (see S1 Appendix). In the initial experiment, the maximum distance agents may move from their home cell (*fradius*) is set to one of the following values: 50 km, 100 km, 150 km, and 200 km. These values reflect the range of Palaeolithic lithic transfer distances described in the archaeological literature and trade distances recorded in the ethnographic literature.

Lithic transfer distances are likely to reflect a combination of individual mobility and trade through social networks. However, if we consider that most flint procurement is embedded in foraging activities (as suggested by [47] p.259) then lithic transfer distances provide a guide to reconstructing individual mobility and thus, the size of annual territories. In a study of Middle and Upper Palaeolithic mobility based on lithic transfers Féblot-Augustin [48](p.237) notes that distances of 40–80 km are common in Western Europe, with a threshold of 125km. Féblot-Augustin concludes that annual territories in Western Europe during the Upper Palaeolithic rarely exceeded a maximum dimension of 100–125 km [48](p.252). Procurement distances > 200 km were very rare in Western Europe (Fig 11 in [48]) but did occur. In a more recent study, Delvigne et al. [49] traced the distribution of flint from a specific flint source in the Paris Basin and report similar figures to Féblot-Augustin, with average transfer distances of 100 km during the Upper Palaeolithic. Regular transfers of up to 200 km occurred but were relatively rare during the LGM, which the authors attribute to the northerly location of the source [49]. Finally, Wiessner [50] documents reciprocal exchanges between hunter-gatherers in the Kalahari over distances of up to 200 km. Based on the figures reported above, we test three possible *fradius* values: 50 km, 100 km, and 200 km.

## Experiments & results

We designed two initial experiments to test the model parameters and make sure they are not producing unexpected results. Experiment 1 tests the impact of the mobility parameter. We used that experiment to design a weighted random-walk pattern of mobility, weighted such that the each cell’s HS value and the distance away from a group are used as the relative probability of that cell being chosen out of all of the cells in the radius being considered (also known as roulette wheel selection and fitness proportionate selection) [51,52]. Experiment 2 tests the impact of different values of the *fradius* parameter. See the supplementary information for a full description of these experiments and their results (S1 Appendix). Following the successful conclusion of experiments 1 and 2, we designed a model to test the impact of habitat suitability on population size and structure, described below.

### The Model: Impact of habitat suitability on survival

Experiments 1 and 2 held the growth rate at 0%, which meant setting the probability of disbanding (the “deathrate”) equal to the probability of creating a new household (the “birthrate”). In both experiments, HS-weighted random walks produced a spatial clustering of agents in high habitat suitability cells resulting in slightly larger populations in these areas. In the final model, we test the impact of habitat suitability (HS) on regional population structure and growth rates and so, in addition to weighting the random walks using HS, we scale the agents’ probability of survival to the HS value of their current cell at each time-step. We expect that groups of agents in more suitable habitats (i.e., regions with relatively high HS values) will experience higher growth rates due to decreased deathrates, while groups in more “risky”, marginal habitats (low HS values) will experience low to negative growth, with knock-on effects on regional population size and structure. There are no archaeological or ethnographic datasets to help us determine what the magnitude of the effect of HS on mortality and thus, population growth rates should be. In this experiment, therefore, we test different magnitudes of effect, observing their impact on overall growth rate. The null hypothesis is that the population’s growth rate should be within the limits described in the archaeological literature, that is between 0 and 0.04% [40,44].

In the final model we assume a simple, linear relationship between the probability of an agent persisting (*deathrate*) and HS. The HS index has a scale of 0 to 1, where 0 is highly unsuitable and 1 is highly suitable. We cannot simply assume that HS 1 = 100% probability of persisting and conversely, that HS 0 = 0% probability of persisting, however, because our null hypothesis is that the growth rate should approach 0% and the *birthrate* (probability of a new agent being created) is fixed to 17.6% / yr (see section 2.2.2, above). Under these conditions, a growth rate ~0% would only occur when HS values approach HS = 1 (which is extremely rare). As a result, the overall growth rate would be far lower than expected. A trial run confirmed that this setting results in total extinction of the metapopulation in a very short time. Therefore, we need to set up a new parameter that allows us to test the impact of HS on survival under conditions consistent with expected growth rates.

**Fig 3:**
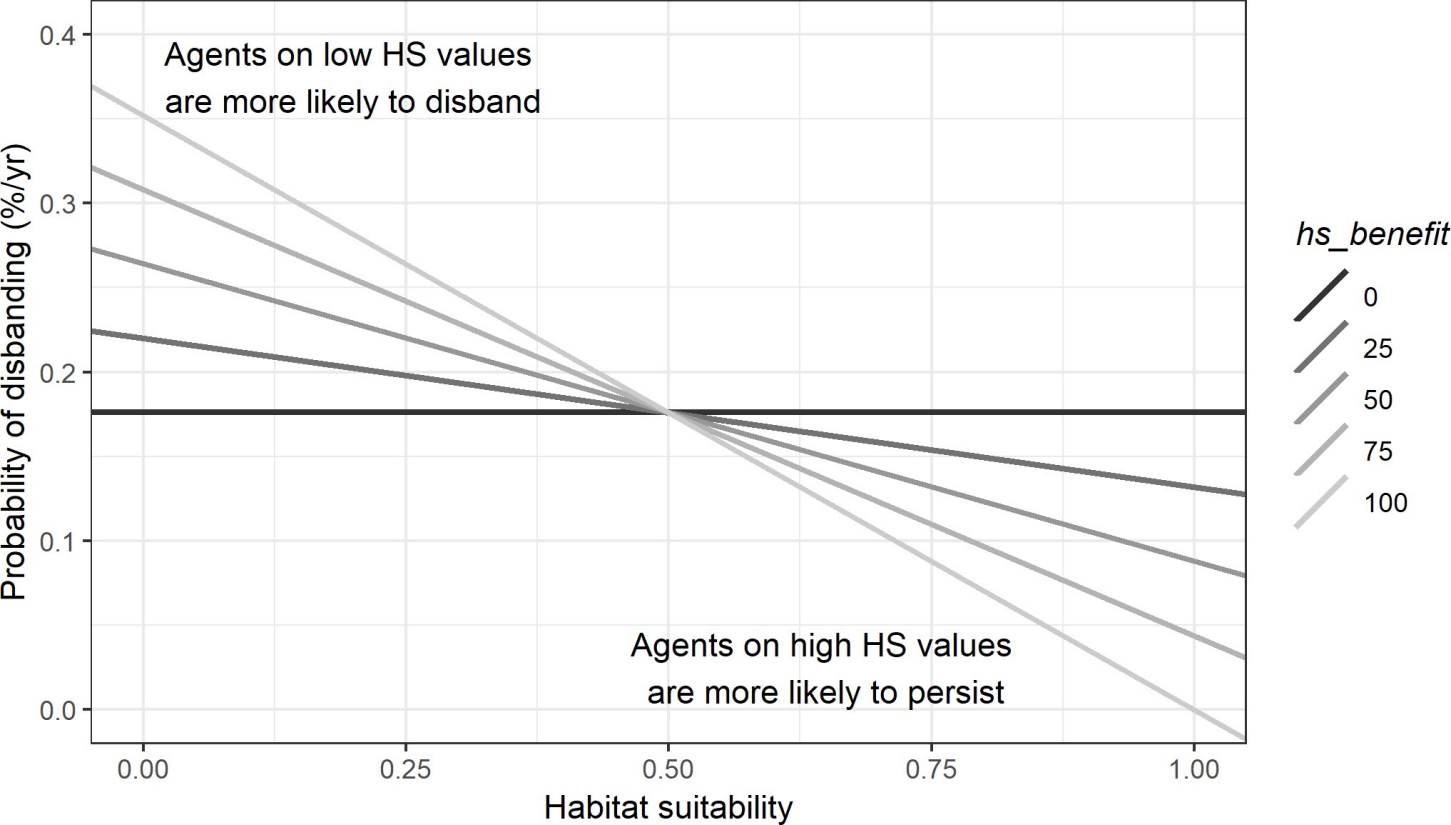
In experiments 1 & 2, habitat suitability had no effect on the deathrate which was set equal to the birthrate. Here, we vary the benefit agents receive from being on higher HS values (and conversely, the penalty for being on cells with lower HS values) using the hs_benefit parameter.

The new model parameter, *hs_benefit*, (Eq 1) creates the relationship:

$$d_{h1.0}=d_{h0.5}-(b*a)$$ (1)

where *b* is the *birthrate*, *a* is *hs_benefit*, *d*_*h0*.*5*_ is the *deathrate* at HS 0.5 and *d*_*h1*.*0*_ is the *deathrate* at HS = 1.0. We set the *deathrate* equal to the *birthrate* at HS = 0.5, which means zero growth occurs on cells at the middle of the HS value range. Using eq.1 we then derive the slope and y-intercept describing the linear relationship between HS and the *deathrate* allowing us to calculate the probability of persistence for all possible HS values (Fig 3).

We use the *hs_benefit* parameter to test a range of possible relationships between the probability of persistence and habitat suitability in 25% increments. Concurrently, we conducted a series of runs with *fradii* values of 50, 100 and 200 km, using HS-weighted mobility. We conducted five runs of each parameter combination and each run was 3000 monthly time steps long, or 250 years. All experiments, including the code which exports the data tables and maps are included in the model code (https://doi.org/10.25937/na38-tj46). The processing time required for larger radii and greater *hs_benefit* runs is very high due to the large number of agents, as we shall see below. For these more computationally expensive runs, we shifted from our standard desktop processing to *Graham*, a high-performance computing system operated by the University of Waterloo.

### The model results

**Fig 4:**
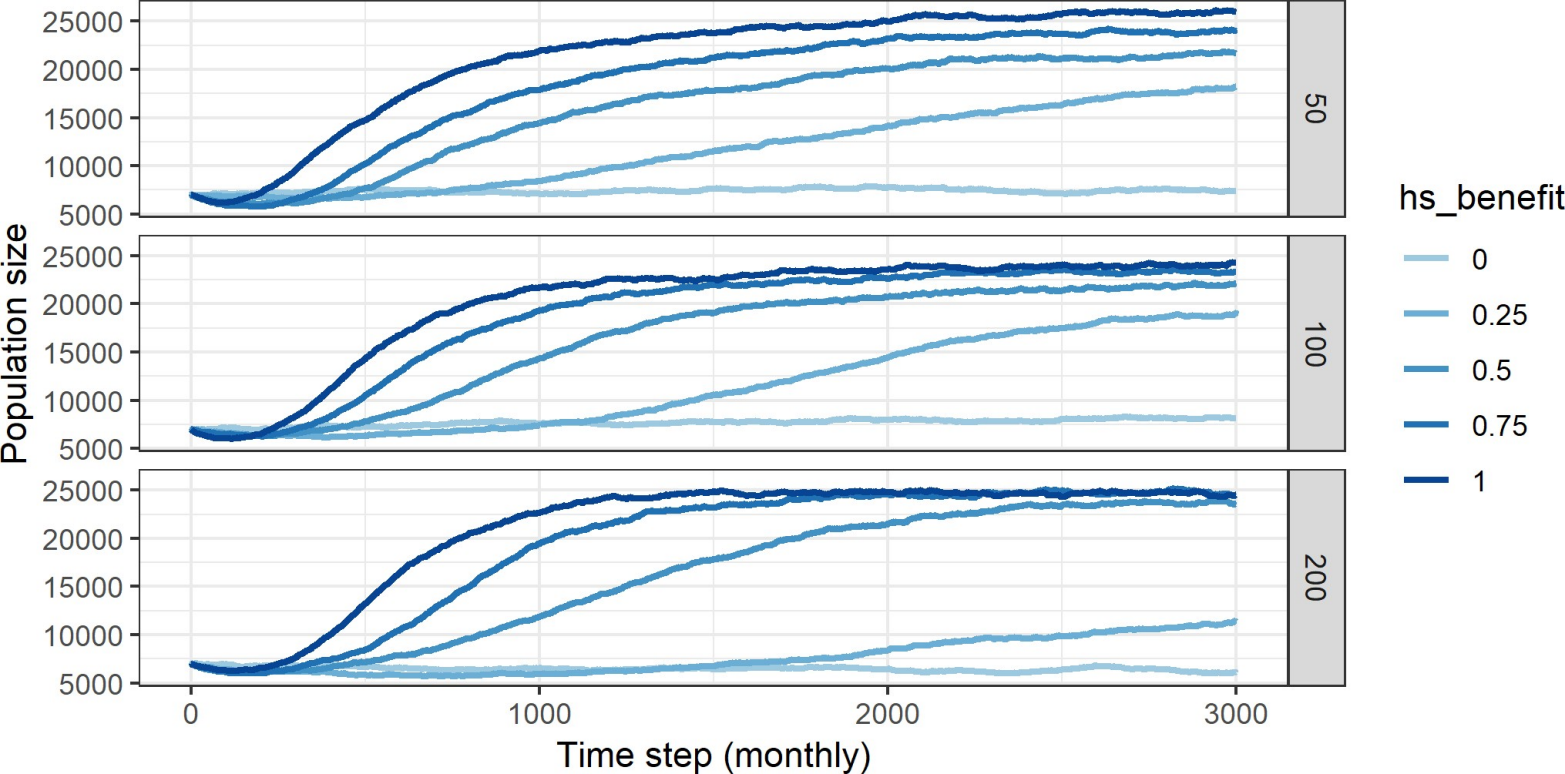
Metapopulation size increase as a function of monthly time steps for five values of hs_benefit at three different scales of mobility.

At the beginning of the runs, the initial metapopulation of 7000 randomly distributed agents suffers a population decline as agents in areas distributed far from core areas suffer attrition. The metapopulation survives within the core regions, and then begins to grow from these core areas in most of the experiments (Fig 4). Note that all R scripts used to evaluate the model outputs and produce the figures below are available for download in our SocArXiv repository (http://doi.org/10.17605/OSF.IO/N24RQ).

Fig 4 tracks the overall metapopulation size for different values of *hs_benefit* and of mobility (fradius) over the 3000 monthly time steps or 250 years of the runs. When *hs_benefit* = 0 the size of the population remains steady since HS does not affect the deathrate, which remains equal to the birthrate and so the growth rate is 0. As *hs_benefit* increases (Fig 4) the population grows at a rate roughly proportional to the magnitude of *hs_benefit*. In several of the experiments the metapopulation reaches the carrying capacity of the environment (~25000 agents) before the end of the run (Fig 4). In the other experiments (*hs_benefit* ≥ 0.25) the population was still growing when the run was terminated and would probably have reached the size threshold eventually. This reflects the fact that model parameters only allow agents to actualise the potential birthrate if they have available space for their “offspring” to move to, effectively treating HS as a proxy for carrying capacity. This has the effect of depressing the overall birthrate in regions with more spatially clustered resources (while maintaining the deathrate steady) independently of *hs_benefit*.

**Fig 5:**
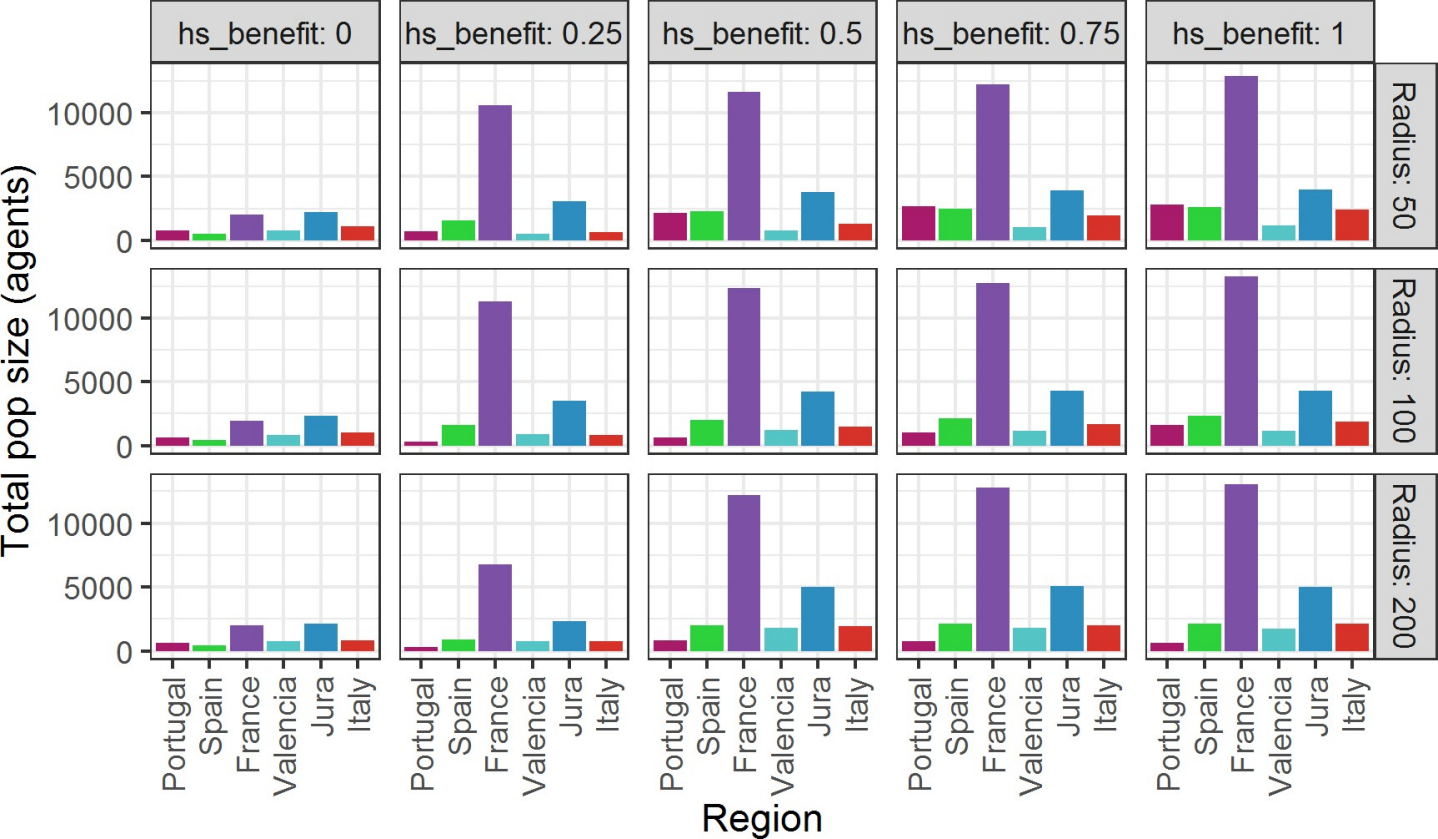
Final population size by “region” for different values of hs_benefit and fradius. Region colors follow legend in the next figure and the map colors in Fig 2.

The model results show that if the structure of the environment (HS) has no impact on the probability of survival the initial population size remains stable. The distribution of the population is only weakly focussed on core areas. Increasing the chances of survival for agents at higher HS cell values (by increasing *hs_benefit > 0*) and conversely, decreasing the chance of survival on low HS cells, increases the size of the metapopulation as agents move onto cells with higher HS values (due to the weighted random walk) where their probability of survival is higher. The metapopulation grows until it reaches carrying capacity (Fig 4) though population growth is not uniformly distributed across the regions (Fig 5).

The final population size of each region reflects the size of the core area, which contains higher HS values (Fig 3A) and, to a lesser extent, the size of the region (Fig 3B) and its average HS value. The Central (France) region, for example, has the largest core area, is the second largest region, and in addition to having the highest average HS score (Table 1), which results in a higher population growth relative to all other regions in all of the experiments. As regional populations grow, they expand from the core area towards the periphery of the region and will, where made possible by the radius of mobility, migrate into adjacent regions over generations of agent movements. The size of a region relative to its core area and the distance between core areas dictates the probability and direction of migration.

**Fig 6:**
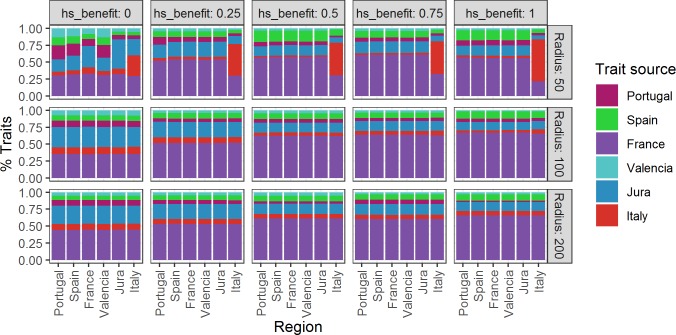
Genetic structure of regional populations for different values of hs_benefit and fradius.

The genetic results (Fig 6) reflect the fact that regions with larger and better connected core areas have a greater chance of growth and subsequent migration. The well-connected Central region contains the largest population which, as it expands outward and migrates, usually comes to dominate the genetic profiles of other regional populations. Relatively higher rates of mortality, compared to the “birth” of new agents, outside the Central region due to lower average HS scores also favour the spread of “French” traits through migration and admixture. Nevertheless, the results also show that gene flow occurs between all of the regions, at all scales of mobility and under all test conditions except when mobility is constrained within a 50 km radius, and even then some gene flow occurs. At this lowest mobility condition of 50 km, Italy retains a larger proportion of its original traits reflecting its relatively isolated geographic position.

**Fig 7:**
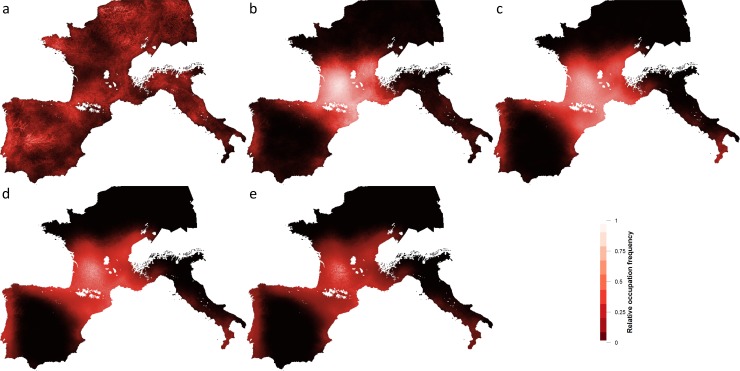
Relative occupation frequency for increasing degrees of hs_benefit with fradius = 50 km. hs_benefit values a) 0, b) 0.25, c) 0.50, d) 0.75, e) 1.0. n.b. Each map's color scheme is normalized to its own maximum occupation frequency value and is exported by the model to represent the final state of one run. European border polygons republished from GADM.org under a CC BY license, with permission from GADM, original copyright 2018.

**Fig 8:**
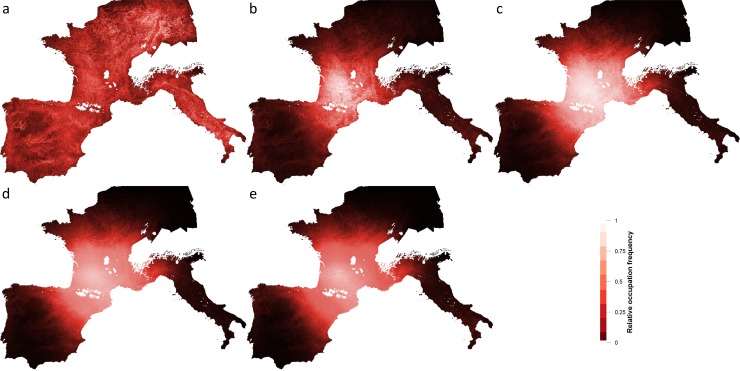
Relative occupation frequency for increasing degrees of hs_benefit with fradius = 200 km. hs_benefit values a) 0, b) 0.25, c) 0.50, d) 0.75, e) 1.0.

The weighted random-walk means that groups occupy much of the landscape occasionally, however, Figs 7 and 8 (below) show the frequency of occupation over the whole domain, which reflects the cumulative effect of higher population growth rates in the core regions. The Central region (the “France” region, which includes northeastern Spain) is the most intensively occupied region during the LGM and therefore reflects the location of a large “glacial refugium”. As higher *hs_benefit* values create a growing imbalance between low and high HS cells, regional disparities in occupation frequencies become more apparent, e.g. central Iberia and northern Europe are eventually abandoned in favour of the Central region when *hs_benefit* = 1.

## Discussion

### Implications of results

We reject the null hypothesis that habitat suitability does not affect rates of survival and thus population structure (*hs_benefit* = 0), because it produces demographic patterns at odds with the archaeological record. For example, it predicts a relatively large proportion of the population would inhabit the “Jura” region (Fig 5) which has a core area in the Jura but includes Central European countries as well as northern latitudes in Western Europe for which there is little or no evidence of human presence during the LGM (Fig 7).

If we accept the hypothesis that habitat suitability reflects a suite of environmental variables that affect human survival, however, we have to determine the magnitude of the effect. We tested different magnitudes of effect through the introduction of a parameter (*hs_benefit*) that modifies the probability of survival as a function of HS. In the absence of robust measures of productivity, it isn’t possible to calculate the carrying capacity of the different regions in our study but the habitat suitability (HS) index serves as a proxy. When HS affects the survival rate through incremental increases in *hs_benefit*, population growth increases until the carrying capacity of the environment is reached, at approximately 25,000 agents or 100,000 individuals, but remains within the upper range of archaeological estimates [33,40,44] in all of our experiments. Different magnitudes of *hs_benefit* are all possible, therefore, and the difference between them is mostly reflected in the speed with which the metapopulation reaches carrying capacity and the concentration of the population towards the core areas.

An *hs_benefit* value of 0.25 is the most “conservative” experiment and when human mobility is constrained to a radius of 50 km under these conditions, regional genetic signatures are evident and Italy retains a strong genetic identity. In subsequent experiments, with increasing penalties for occupying cells with low HS values, the metapopulation expands but the regional genetic signatures become more and more homogeneous at all levels of mobility, with the notable exception of Italy at the 50 km radius. At the same time, the level of human activity in regions beyond the core areas, e.g. northern Europe and central Iberia, diminishes (Fig 7 and Fig 8).

Under all of the test conditions where HS has an impact on human survival, the Central region (which includes France and northeastern Spain) has the largest population by far and several of the other regions struggle to maintain themselves. It is interesting to note that the Jura contains a fairly large population, which is not well reflected in the archaeological literature, although this region is receiving renewed attention [53–56]. Gene flow occurs between all of the regions, in all of the experiments, but genes associated with the Central “French” region dominate the regional genetic profiles. The dominance of the “French” genetic signature is due to its population size (which is determined by the size of its core area) and high connectivity with several adjacent core regions, which means that the expanding population has room to grow. This trend becomes more pronounced as the strength of the effect of HS on survivorship is increased, i.e., as the structure of the environment takes on a greater role in determining the chances of survival.

Our results suggest the important role the Central region would have played as a glacial refugium during the LGM. Its large core area is capable of sustaining a large population and leads to net population growth even as other regions experience more agent “deaths” than “births”. The strength of this effect increases as habitat suitability is given a greater role in determining the probability of survival (i.e. with increasing *hs_benefit*).

We interpret the results of these experiments in terms of a “source” and “sink” dynamic, similar to the one proposed for the Middle Pleistocene of Europe [57]. From this perspective, the Central region acts as the “source” of population growth, due to its centrality (connectedness), its large core area and its relatively high habitat suitability index. The other regions appear to have acted as “sinks”, with a net population loss being replenished by in-migration from the Central region. The genetic signature from “Valencia” (Mediterranean Spain), for example, disappears in two of our scenarios (Fig 6) albeit the least conservative of the *hs_benefit* scenarios. The source-sink dynamic has important implications for the genetic and cultural diversity of people living in Europe during the LGM.

We test three ranges of mobility (50 km, 100 km and 200 km). While lithic transfer patterns suggest mobility of up to 200 km, we suspect that movement on this scale would have been relatively rare, possibly falling into the category of “non-utilitarian” mobility described by Whallon [58]. Furthermore, with a radius of 200 km the implied territory size is twice as large as the highest value recorded for ethnographically known hunter-gatherer groups (Table 4–1 in [41]). It is possible that regions with more spatially dispersed patches of resources required higher levels of mobility, but the shorter ranges are probably better approximations of average mobility patterns during the LGM. A range of mobility of 50 km correspond to average lithic transfer distances recorded for Western Europe during the Upper Paleolithic [48] and produces annual territories of 7,854 km^2^. This figure is compatible with the size of annual territories reported for hunter-gatherers who depend upon meat as an important resource [41]. Regional genetic variability is highest when mobility is restricted to a 50 km radius particularly in Italy, as one would expect given its poor connectivity with other regions. Since we did not include the Balkans in this study we cannot speak to possible gene flow from this region, however. When mobility is constrained to 50 km, land-use patterns concentrate around core areas and the “hinterlands” are abandoned, whereas at 100 and 200 km people still make use of these areas, although occupation intensity focusses on the core areas (compare Fig 8A and 8B). There is evidence in the archeological literature that regions outside of the traditional refugia, such as the Paris Basin [59,60] and Central Spain [61,62] could have been exploited during the LGM which could demonstrate the importance of at least occasional long-distance movements.

### Genetics and material culture of the Last Glacial Maximum

Ancient DNA (aDNA) from human fossils dating to the LGM has the potential to validate or invalidate the conclusions we make in this paper. Indeed, our hypothesis (that HS affects the population dynamics of Western Europe during the LGM) and the ensuing models lead us to make very concrete predictions about regional population size, the genomic diversity and the structure of coalescent trees that researchers could construct from new samples taken from across European sites dating to the LGM. These include the existence of gene flow between all of the core areas within Western Europe, the dominance of genes from the Central region (including France and Northeastern Spain) which acts as a source population for Western Europe, and in some scenarios, the loss of genetic diversity from Mediterranean Spain (“Valencia”). In a scenario involving low human mobility patterns, Italy retains a distinct regional genetic signature.

Despite the increasingly rapid pace of sequencing, the LGM has been largely neglected so far [63] hampering our ability to test our predictions. Morphological data suggest that the human gene pool changed during the LGM [64]. Fu et al. [65] discuss genetic transitions surrounding the LGM at a continental scale, but most of their samples date to well before or well after the LGM and therefore cannot be used to evaluate the metapopulation structure during the LGM itself. Indeed, the only European genome of relevance is an individual from El Miron Cave, Spain dated to shortly after the LGM at ~18.7 ka and associated with the Magdalenian industry. Fu et al. [65] conclude, from comparing this genome to older European Gravettian genomes from ~34–26 ka and younger Magdalenian genomes from ~19–14 ka, that an endogenous European population survived the LGM within Europe and repopulated the continent afterwards. Similarly, Posth et al. [17] find a significant population contraction during the European LGM due to the loss of mtDNA haplogroup M in the post-LGM metapopulation, but they do not present genomes dating to the LGM itself. A greater number of ancient genomes, with finer spatial and temporal granularity, will be needed to test the predictions presented in this paper.

Our model does not include social or linguistic variables, nor do we examine cultural diffusion. However, our results have implications for cultural evolution. Proponents of the Cumulative Culture hypothesis have pointed out that demographic processes affect cultural transmission and rates of cultural innovation. For example, Powell et al. [4] show that levels of cultural innovation increase with the number and level of interaction between regional populations. Similarly, Derex and colleagues show that innovation rates may accelerate when the metapopulation is at an intermediate level of connectivity [66,67]. These studies and others suggest that the large and well-connected metapopulation we predict for the Central region will have favoured cultural transmission and made this region an important hub for cultural innovation. This prediction is compatible with the archaeological record. The Gravettian technocomplex which pre-existed the LGM was displaced by the Solutrean culture, which overlaps with the Early Magdalenian and Badegoulian in the Central region [68] and the Epigravettian in Italy [69]. The occupation frequencies predicted by the model do not suggest a direct connection between the core areas in the Iberian Peninsula (Atlantic and Mediterranean) but rather, suggest that these regions were indirectly connected through the central “French” region which may explain the cultural differences between these regions [18,70]. In the space of ~4,000 years, the Gravettian technocomplex, which was distributed across Western Europe prior to the LGM, is succeeded by the Solutrean in France and the Iberian Peninsula, which overlaps with the Early Magdalenian and the Badegoulian in France around 23,500 cal. BP [68]. The Epigravettian persists in Italy throughout the LGM [69].

Our results also predict that, unless we accept high levels of mobility and annual territories with radii of 100 km or 200 km, and high selective pressure (*hs_benefit* = 1), Italy forms a regionally distinct part of the Western European metapopulation, both in terms of its size and genetic composition. This observation is perfectly compatible with the material culture record, during the LGM the Epigravettian arose in Italy, distinguishing it from the other regions [69]. We will explore the dynamics of cultural interaction further in an upcoming paper.

## Conclusions

The goal of this research is to test the impact of habitat suitability [1] on the demographic and genetic structure of human populations living in Western Europe during the LGM. Habitat suitability is an index defined by a combination of topographic (elevation and slope) and seasonal climate variables, with emphasis on variability.

The initial conditions of the model place agents randomly in each region. A weighted random walk function then allows them to relocate to areas with higher HS values within a set distance (radius) from their “home” cell (50 km, 100 km, 200 km). The choice of test radii reflects estimates of Palaeolithic mobility reported in the literature. The base probability of agents disbanding (the “deathrate”) is calculated as a function of the “birthrate” (itself based on ethnographic information) to produce zero population growth (the null hypothesis) which also conforms to archaeologically derived estimates of Palaeolithic growth rates. We then experiment with different levels of selective pressure by introducing a new parameter (*hs_benefit*) which alters survival rates as a function of HS.

The results show that during the LGM, Western Europe contained six core areas (Fig 2A) that had a high frequency of occupation (Fig 7 and Fig 8) and are composed of clusters of highly suitable habitat. The core areas are connected to each other directly (through a shared border) or indirectly through the Central region. We reject the null hypothesis (no selective pressure resulting from HS) on the basis of the unrealistic predictions it makes about the spatial distribution of the metapopulation. Total population sizes resulting from all other experiments reach the carrying capacity predicted by the model, approximately 25,000 agents or 100,000 individuals. Regional population size (i.e., the size of the population that lives in a given core area or its periphery) varies with the size of the core area and its mean HS value. The Central region (which includes France and northeastern Spain) has the largest population size in all of our experiments and is connected directly to all of the other regions. As a result, although gene flow occurs between all of the regions, the Central region dominates the genetic profiles of the regional populations. Indeed, the high rate of births compared to deaths in the Central region results in out-migration to the other regions that sustains those populations in a source–sink dynamic. In several experiments, especially at low mobility levels, Italy retains a distinct genetic signature largely due to its relative geographic isolation. Some of the smaller core areas (e.g. Mediterranean Spain) may have struggled to maintain viable populations and thus, their genetic signature may have been lost.

Finally, the spatial structure of the HS landscape suggests demographic patterns that potentially explain the cultural florescence of technology and parietal art forms observed in the archaeological record of the LGM in the Central region.

## Supporting information

S1 Appendix Initial experiments and expanded methods. Preliminary experiments to establish proper functioning of the model, detailed description of agent-based model using the ODD Protocol, and expanded step-by-step explanation of the GIS methodology involved in the creation of regions and core areas.
(PDF)

## References

[1] Burke A, Kageyama M, Latombe G, Fasel M, Vrac M, Ramstein G, et al Risky business: The impact of climate and climate variability on human population dynamics in Western Europe during the Last Glacial Maximum. Quaternary Science Reviews. 2017;164: 217–229. 10.1016/j.quascirev.2017.04.001

[2] Derex M, Beugin M-P, Godelle B, Raymond M. Experimental evidence for the influence of group size on cultural complexity. Nature. 2013;503: 389–391. 10.1038/nature12774 24226775

[3] Lehmann L, Aoki K, Feldman MW. On the number of independent cultural traits carried by individuals and populations. Philosophical Transactions of the Royal Society of London B: Biological Sciences. 2011;366: 424–435. 10.1098/rstb.2010.0313 21199846 PMC3013478

[4] Powell A, Shennan S, Thomas MG. Late Pleistocene demography and the appearance of modern human behavior. Science. 2009;324: 1298–1301. 10.1126/science.1170165 19498164

[5] Peltier WR, Fairbanks RG. Global glacial ice volume and Last Glacial Maximum duration from an extended Barbados sea level record. Quaternary Science Reviews. 2006;25: 3322–3337. 10.1016/j.quascirev.2006.04.010

[6] Rasmussen SO, Bigler M, Blockley SP, Blunier T, Buchardt SL, Clausen HB, et al A stratigraphic framework for abrupt climatic changes during the Last Glacial period based on three synchronized Greenland ice-core records: refining and extending the INTIMATE event stratigraphy. Quaternary Science Reviews. 2014;106: 14–28. 10.1016/j.quascirev.2014.09.007

[7] Waelbroeck C, Labeyrie L, Michel E, Duplessy JC, McManus JF, Lambeck K, et al Sea-level and deep water temperature changes derived from benthic foraminifera isotopic records. Quaternary Science Reviews. 2002;21: 295–305. 10.1016/S0277-3791(01)00101-9

[8] Bartlein PJ, Harrison SP, Brewer S, Connor S, Davis BAS, Gajewski K, et al Pollen-based continental climate reconstructions at 6 and 21 ka: a global synthesis. Clim Dyn. 2011;37: 775–802. 10.1007/s00382-010-0904-1

[9] Helmens KF. The Last Interglacial–Glacial cycle (MIS 5–2) re-examined based on long proxy records from central and northern Europe. Quaternary Science Reviews. 2014;86: 115–143. 10.1016/j.quascirev.2013.12.012

[10] Kageyama M, Nebout NC, Sepulchre P, Peyron O, Krinner G, Ramstein G, et al The Last Glacial Maximum and Heinrich Event 1 in terms of climate and vegetation around the Alboran Sea: a preliminary model-data comparison. Comptes Rendus Geoscience. 2005;337: 983–992. 10.1016/j.crte.2005.04.012

[11] Kageyama M, Laîné A, Abe-Ouchi A, Braconnot P, Cortijo E, Crucifix M, et al Last Glacial Maximum temperatures over the North Atlantic, Europe and western Siberia: a comparison between PMIP models, MARGO sea–surface temperatures and pollen-based reconstructions. Quaternary Science Reviews. 2006;25: 2082–2102. 10.1016/j.quascirev.2006.02.010

[12] Kageyama M, Braconnot P, Bopp L, Caubel A, Foujols M-A, Guilyardi E, et al Mid-Holocene and Last Glacial Maximum climate simulations with the IPSL model—part I: comparing IPSL_CM5A to IPSL_CM4. Clim Dyn. 2013;40: 2447–2468. 10.1007/s00382-012-1488-8

[13] Banks WE, d’Errico F, Zilhão J. Human–climate interaction during the Early Upper Paleolithic: testing the hypothesis of an adaptive shift between the Proto-Aurignacian and the Early Aurignacian. Journal of Human Evolution. 2013;64: 39–55. 10.1016/j.jhevol.2012.10.001 23245623

[14] Gamble C, Davies W, Pettitt P, Richards M. Climate change and evolving human diversity in Europe during the last glacial. Phil Trans R Soc Lond B. 2004;359: 243–254. 10.1098/rstb.2003.1396 15101580 PMC1693315

[15] Stewart JR, Stringer CB. Human Evolution Out of Africa: The Role of Refugia and Climate Change. Science. 2012;335: 1317–1321. 10.1126/science.1215627 22422974

[16] Burke A, Levavasseur G, James PMA, Guiducci D, Izquierdo MA, Bourgeon L, et al Exploring the impact of climate variability during the Last Glacial Maximum on the pattern of human occupation of Iberia. Journal of Human Evolution. 2014; 10.1016/j.jhevol.2014.06.003 25034085

[17] Posth C, Renaud G, Mittnik A, Drucker DG, Rougier H, Cupillard C, et al Pleistocene Mitochondrial Genomes Suggest a Single Major Dispersal of Non-Africans and a Late Glacial Population Turnover in Europe. Current Biology. 2016;26: 827–833. 10.1016/j.cub.2016.01.037 26853362

[18] Straus LG, Bicho N, Winegardner AC. The Upper Palaeolithic settlement of Iberia: first-generation maps. Antiquity. 2000;74: 553–566. 10.1017/S0003598X00059913

[19] Straus LG. The upper paleolithic of Cantabrian Spain. Evolutionary Anthropology: Issues, News, and Reviews. 2005;14: 145–158.

[20] Renard C. Continuity or discontinuity in the Late Glacial Maximum of south-western Europe: the formation of the Solutrean in France. World Archaeology. 2011;43: 726–743. 10.1080/00438243.2011.624789

[21] Braconnot P, Harrison SP, Kageyama M, Bartlein PJ, Masson-Delmotte V, Abe-Ouchi A, et al Evaluation of climate models using palaeoclimatic data. Nature Climate Change. 2012;2: 417–424. 10.1038/nclimate1456

[22] van Andel TH. The Climate and Landscape of the Middle Part of the Weichselian Glaciation in Europe: The Stage 3 Project. Quaternary Research. 2002;57: 2–8. 10.1006/qres.2001.2294

[23] van Andel TH, Davies W. Neanderthals and modern humans in the European landscape during the last glaciation: archaeological results of the Stage 3 Project. McDonald Institute for Archaeological Research monographs; 2003.

[24] Vrac M, Marbaix P, Paillard D, Naveau P. Non-linear statistical downscaling of present and LGM precipitation and temperatures over Europe. Climate of the Past. 2007;3: 669–682.

[25] Levavasseur G, Vrac M, Roche DM, Paillard D, Martin A, Vandenberghe J. Present and LGM permafrost from climate simulations: contribution of statistical downscaling. Climate of the Past. 2011;7: 1225–1246. 10.5194/cp-7-1225-2011

[26] Latombe G, Burke A, Vrac M, Levavasseur G, Dumas C, Kageyama M, et al Comparison of spatial downscaling methods of general circulation model results to study climate variability during the Last Glacial Maximum. Geoscientific Model Development. 2018;11: 2563–2579. 10.5194/gmd-11-2563-2018

[27] Vita-Finzi C, Higgs ES. Prehistoric economy in the Mount Carmel area of Palestine: site catchment analysis. Proceedings of the Prehistoric Society. 1970;36: 1–37.

[28] Banks WE, d’Errico F, Peterson AT, Vanhaeren M, Kageyama M, Sepulchre P, et al Human ecological niches and ranges during the LGM in Europe derived from an application of eco-cultural niche modeling. J Archaeol Sci. 2008;35: 481–491. 10.1016/j.jas.2007.05.011

[29] Banks WE, Zilhão J, d’Errico F, Kageyama M, Sima A, Ronchitelli A. Investigating links between ecology and bifacial tool types in Western Europe during the Last Glacial Maximum. Journal of Archaeological Science. 2009;36: 2853–2867. 10.1016/j.jas.2009.09.014

[30] Banks WE, Aubry T, d’Errico F, Zilhão J, Lira-Noriega A, Townsend Peterson A. Eco-cultural niches of the Badegoulian: Unraveling links between cultural adaptation and ecology during the Last Glacial Maximum in France. Journal of Anthropological Archaeology. 2011;30: 359–374. 10.1016/j.jaa.2011.05.003

[31] Gautney JR, Holliday TW. New estimations of habitable land area and human population size at the Last Glacial Maximum. Journal of Archaeological Science. 2015;58: 103–112. 10.1016/j.jas.2015.03.028

[32] Maier A, Lehmkuhl F, Ludwig P, Melles M, Schmidt I, Shao Y, et al Demographic estimates of hunter–gatherers during the Last Glacial Maximum in Europe against the background of palaeoenvironmental data. Quaternary International. 2016;425: 49–61. 10.1016/j.quaint.2016.04.009

[33] Tallavaara M, Luoto M, Korhonen N, Järvinen H, Seppä H. Human population dynamics in Europe over the Last Glacial Maximum. PNAS. 2015;112: 8232–8237. 10.1073/pnas.1503784112 26100880 PMC4500234

[34] Harrison SP, Bartlein PJ, Prentice IC. What have we learnt from palaeoclimate simulations? Journal of Quaternary Science. 2016;31: 363–385. 10.1002/jqs.2842

[35] Fitzhugh B, Phillips SC, Gjesfjeld E. Modeling Variability in Hunter-Gatherer Information Networks: An Archaeological Case Study from the Kuril Islands In: Whallon R, Lovis WA, Hitchcock RK, editors. Information and its role in hunter-gatherer bands. Los Angeles: UCLA/Cotsen Institute of Archaeology Press; 2011 pp. 85–115.

[36] Whallon R, Lovis WA, Hitchcock RK. Information and its role in hunter-gatherer bands. Los Angeles: UCLA/Cotsen Institute of Archaeology Press; 2011.

[37] Barton CM, Riel-Salvatore J. Agents of change: modeling biocultural evolution in upper pleistocene western eurasia. Advances in Complex Systems. 2012;15: 1150003 10.1142/S0219525911003359

[38] Hopkinson T. The Transmission of Technological Skills in the Palaeolithic: Insights from Metapopulation Ecology In: Roberts BW, Linden MV, editors. Investigating Archaeological Cultures. New York: Springer; 2011 pp. 229–244. 10.1007/978-1-4419-6970-5_12

[39] Ord JK, Getis A. Local Spatial Autocorrelation Statistics: Distributional Issues and an Application. Geographical Analysis. 1995;27: 286–306. 10.1111/j.1538-4632.1995.tb00912.x

[40] Bocquet-Appel J-P, Demars P-Y, Noiret L, Dobrowsky D. Estimates of Upper Palaeolithic meta-population size in Europe from archaeological data. Journal of Archaeological Science. 2005;32: 1656–1668. 10.1016/j.jas.2005.05.006

[41] Kelly RL. The lifeways of hunter-gatherers: the foraging spectrum. 2nd ed Cambridge: Cambridge University Press; 2013.

[42] Barton CM, Riel-Salvatore J, Anderies JM, Popescu G. Modeling Human Ecodynamics and Biocultural Interactions in the Late Pleistocene of Western Eurasia. Hum Ecol. 2011;39: 705–725. 10.1007/s10745-011-9433-8

[43] Pennington R. Hunter-gatherer demography. Cambridge, UK: Cambridge University Press; 2001.

[44] Zahid HJ, Robinson E, Kelly RL. Agriculture, population growth, and statistical analysis of the radiocarbon record. PNAS. 2016;113: 931–935. 10.1073/pnas.1517650112 26699457 PMC4743794

[45] Gurven M, Kaplan H. Longevity Among Hunter- Gatherers: A Cross-Cultural Examination. Population and Development Review. 2007;33: 321–365. 10.1111/j.1728-4457.2007.00171.x

[46] Bettinger RL. Prehistoric hunter–gatherer population growth rates rival those of agriculturalists. PNAS. 2016;113: 812–814. 10.1073/pnas.1523806113 26772312 PMC4743830

[47] Binford LR. Organization and formation processes: looking at curated technologies. Journal of anthropological research. 1979;35: 255–273.

[48] Féblot-Augustins J. La mobilité des groupes paléolithiques. Bulletins et Mémoires de la Société d’anthropologie de Paris. 1999;11: 219–260.

[49] Delvigne V, Fernandes P, Piboule M, Lafarge A, Raynal J-P. Circulation de géomatières sur de longues distances au Paléolithique supérieur: le cas des silex du Turonien du Sud du Bassin parisien. Comptes Rendus Palevol. 2017;16: 82–102. 10.1016/j.crpv.2016.04.005

[50] Risk Wiessner P., reciprocity and social influences on! Kung San economics. Politics and history in band societies. 1982;61: 84.

[51] Payette N. NetLogo Rnd-Extension [Internet]. 2013 Available: https://github.com/NetLogo/Rnd-Extension

[52] Lipowski A, Lipowska D. Roulette-wheel selection via stochastic acceptance. Physica A: Statistical Mechanics and its Applications. 2012;391: 2193–2196. 10.1016/j.physa.2011.12.004

[53] Cupillard C, Fornage S, Malgarini R. Le Paléolithique supérieur ancien dans le quart nord-est de la France: l’exemple de la Franche-Comté. Société préhistorique française. 2009;LVI: 351–363.

[54] Cupillard C, Magny M, Bocherens H, Bridault A, Bégeot C, Bichet V, et al Changes in ecosystems, climate and societies in the Jura Mountains between 40 and 8 ka cal BP. Quaternary International. 2015;378: 40–72. 10.1016/j.quaint.2014.05.032

[55] Demars P-Y. Changements climatiques et occupation de l’espace. Les derniers chasseurs cueilleurs d’Europe face à la déglaciation.[Climatic changes and settlement patterns. The last prehistoric hunter-gatherers of Europe confronted with the déglaciation]. Quaternaire. 2002;13: 289–296.

[56] Terberger T, Street M. New evidence for the chronology of the Aurignacian and the question of Pleniglacial settlement in western central Europe. The Chronology of the Aurignacian and of the Transitional Technocomplexes: Dating, Stratigraphies, Cultural Implications, Trabalhos de Arqueologia. 2003;33: 213–221.

[57] Dennell RW, Martinón-Torres M, Bermúdez de Castro JM. Hominin variability, climatic instability and population demography in Middle Pleistocene Europe. Quaternary Science Reviews. 2011;30: 1511–1524. 10.1016/j.quascirev.2009.11.027

[58] Whallon R. Social networks and information: Non-“utilitarian” mobility among hunter-gatherers. Journal of Anthropological Archaeology. 2006;25: 259–270. 10.1016/j.jaa.2005.11.004

[59] Debout G, Olive M, Bignon O, Bodu P, Chehmana L, Valentin B. The Magdalenian in the Paris Basin: New results. Quaternary International. 2012;272–273: 176–190. 10.1016/j.quaint.2012.05.016

[60] Hinguant S, Biard M. Le Paléolithique supérieur ancien de la vallée de l’Erve (Mayenne): un état des connaissances In: Bodu P, Chehmana L, Klaric L, Mevel L, Soriano S, Teyssandier N, editors. Le Paléolithique supérieur ancien de l’Europe du Nord-Ouest. Société préhistorique française; 2013 pp. 15–18.

[61] Alcaraz-Castaño M, López-Recio M, Tapias F, Cuartero F, Baena J, Ruiz-Zapata B, et al The human settlement of Central Iberia during MIS 2: New technological, chronological and environmental data from the Solutrean workshop of Las Delicias (Manzanares River valley, Spain). Quaternary International. 2017;431: 104–124. 10.1016/j.quaint.2015.06.069

[62] Yravedra J, Julien M-A, Alcaraz-Castaño M, Estaca-Gómez V, Alcolea-González J, de Balbín-Behrmann R, et al Not so deserted…paleoecology and human subsistence in Central Iberia (Guadalajara, Spain) around the Last Glacial Maximum. Quaternary Science Reviews. 2016;140: 21–38. 10.1016/j.quascirev.2016.03.021

[63] Skoglund P, Mathieson I. Ancient Genomics of Modern Humans: The First Decade. Annual Review of Genomics and Human Genetics. 2018;19: 381–404. 10.1146/annurev-genom-083117-021749 29709204

[64] Niskanen M, Ruff CB, Holt B, Sládek V, Berner M. Temporal and Geographic Variation in Body Size and Shape of Europeans from the Late Pleistocene to Recent Times In: Ruff CB, editor. Skeletal Variation and Adaptation in Europeans. Hoboken, NJ, USA: John Wiley & Sons, Inc; 2017 pp. 49–89. 10.1002/9781118628430.ch4

[65] Fu Q, Posth C, Hajdinjak M, Petr M, Mallick S, Fernandes D, et al The genetic history of Ice Age Europe. Nature. 2016;advance online publication. 10.1038/nature17993 27135931 PMC4943878

[66] Derex M, Perreault C, Boyd R. Divide and conquer: intermediate levels of population fragmentation maximize cultural accumulation. Phil Trans R Soc B. 2018;373: 20170062 10.1098/rstb.2017.0062 29440527 PMC5812974

[67] Derex M, Boyd R. Partial connectivity increases cultural accumulation within groups. PNAS. 2016;113: 2982–2987. 10.1073/pnas.1518798113 26929364 PMC4801235

[68] Ducasse S. What is left of the Badegoulian “interlude”? New data on cultural evolution in southern France between 23,500 and 20,500 cal. BP. Quaternary International. 2012;272–273: 150–165. 10.1016/j.quaint.2012.05.018

[69] Blockley S, Pellegrini M, Colonese AC, Lo Vetro D, Albert PG, Brauer A, et al Dating human occupation and adaptation in the southern European last glacial refuge: The chronostratigraphy of Grotta del Romito (Italy). Quaternary Science Reviews. 2018;184: 5–25. 10.1016/j.quascirev.2017.09.007

[70] Straus LG, González Morales MR. The Magdalenian settlement of the Cantabrian region (Northern Spain): The view from El Miron Cave. Quaternary International. 2012;272–273: 111–124. 10.1016/j.quaint.2012.03.053

