# Supplementary material for: Habitat suitability and the genetic structure of human populations during the Last Glacial Maximum (LGM) in Western Europe
Source: PLoS One. 2019 Jun 19;14(6):e0217996. doi: 10.1371/journal.pone.0217996 (PMC6583941; doi:10.1371/journal.pone.0217996)
Supplement: S1 Appendix — Preliminary experiments to establish proper functioning of the model, detailed description of agent-based model using the ODD Protocol, and expanded step-by-step explanation of the GIS methodology involved in the creation of regions and core areas. (PDF) [file pone.0217996.s001.pdf]

Supplementary Information

Part 1: Initial Experiments & Results

Experiment 1: Random versus targeted mobility

In our first experiment, we compare two possible mobility patterns, both based on a weighted random walk algorithm but with different factors within the weightings. In the first pattern the weighting is simply based on distance (inverse-distance weighting). In the second pattern, weighting is based on distance and the habitat suitability index (see main text). This gives agents the capacity to use their knowledge of the landscape to make informed decisions, something we've previously termed spatial foresight [1,2]. For this experiment, we hold birth and deathrates constant, i.e. the population growth rate is zero and agents have an equal probability of disbanding or generating a new household irrespective of the habitat suitability of the cells on which they "live" at each time step. We set the search radius to an intermediate value of 100 km and then conduct two sets of model runs. Each run lasts 3000 time steps or 250 years and we conduct 10 runs for each set, averaging the results. In the first set agents move randomly to any cell within their search radius at every time step, with a higher probability of moving to closer cells (i.e., inverse distance-weighted mobility). In the second set of runs, the weighting is based on two factors: distance and the habitat suitability index (HS). In other words, more weight is given to high HS values, mitigated by distance (see model description below for details).

This experiment allows us to test whether the mobility pattern we adopt in the following experiments functions as expected.

Experiment 1: Results

The regional population sizes observed (Fig. S1a & b) reflect the size of the regions, which conditions the initial allocation of randomly generated agents on the landscape (see Fig. 2c). This is as expected since all agents, irrespective of the HS value of the cells they occupy, have an equal probability of disbanding or producing new agents.

The results show that, when mortality rates are unaffected by habitat suitability, random mobility patterns weighted using: 1) distance alone, and; 2) a combination of distance and habitat suitability produce similar results, i.e., regional population sizes are consistent with the size of the region. In other words, weighting mobility in favour of higher HS indices doesn't introduce unexpected biases into the model.

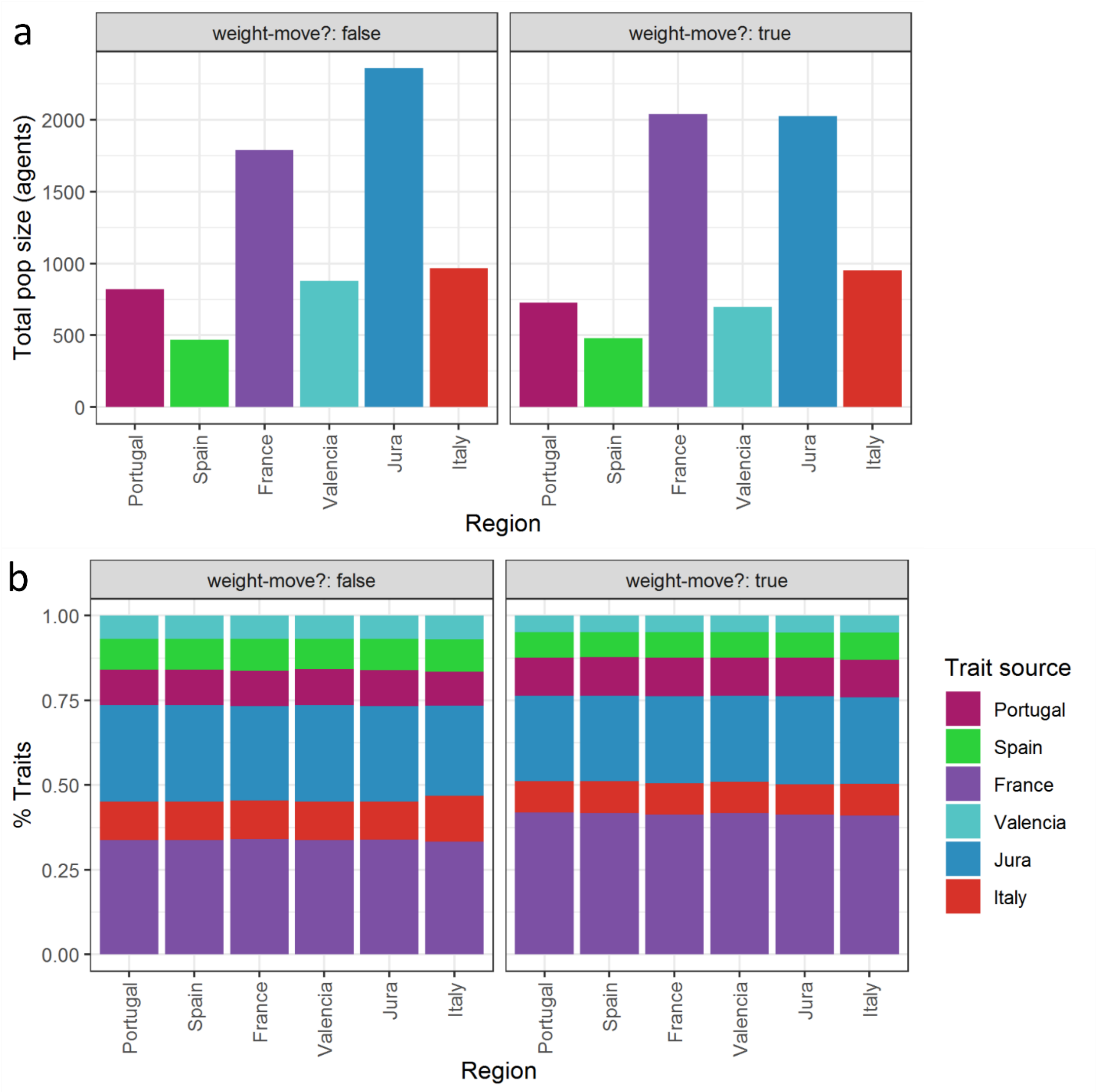

Fig. S1. a) shows the counts of agents in each of the contiguous region areas (ignoring their genomic trait frequencies) and b) shows the averaged genetic percentages among the agents in each region.

However, differences arise when we compare the summed occupation history of the agents across the landscape (Fig. S2). In this figure, we can see that the weighted random walk that includes habitat suitability increases the concentration of agents in cells with higher habitat suitability values over time. So, while regional population sizes remain relatively stable, the occupation of the landscape varies considerably within each region when mobility is weighted using HS. For example, in a distance-weighted only run the population of the Iberian Peninsula occupies the landscape relatively evenly, whereas in a distance and HS-weighted run,

occupations concentrate on the northern and western coasts of the Peninsula. This demonstrates that our model is functioning as it should when we adopt an HS-weighted mobility pattern, as we will in subsequent experiments.

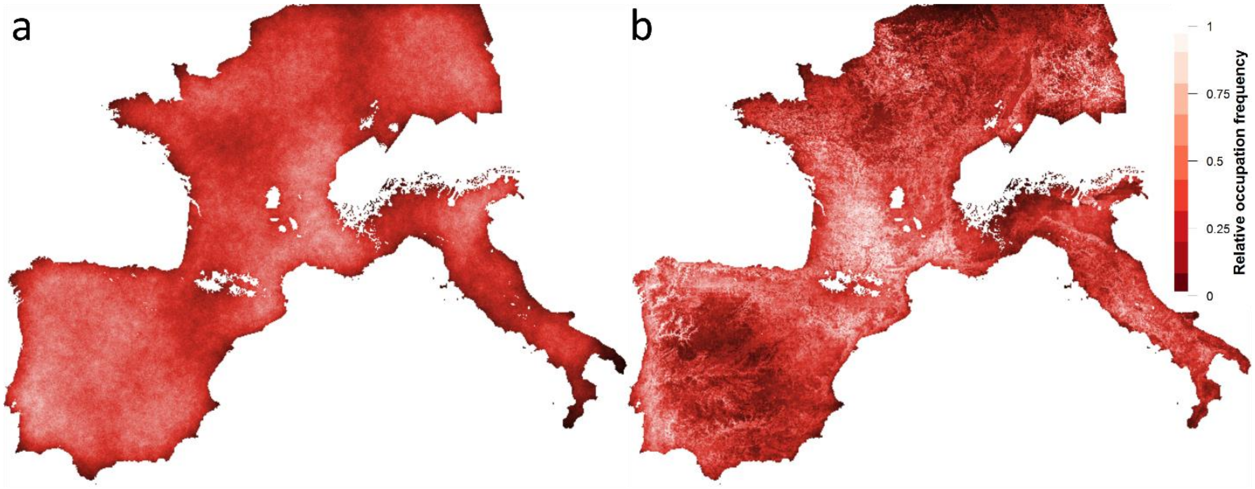

Fig. S2. Relative occupation frequency represents a per cell count of the number of months an agent was present. Occupation frequency for a) a run with mobility a distance-weighted random walk, and b) a run with mobility also weighted to the habitat suitability index. N.b. each map's colour scheme is normalized to its own occupation frequency distribution using a contrast stretch (mean +- 3 sd) and represents just one sample run, not an average over multiple runs.

Experiment 2: Annual territory size

In the next experiment we want to establish the impact of a range of different annual foraging radii (a proxy for annual territory size) when we weight mobility using both distance and habitat suitability.

We experiment with 50, 100, 150, and 200 km as our agent's maximum foraging radii based on the ethnographic and archaeological data cited above (Fig. S3). As noted in the description of Experiment 1, above, agent movement is inverse distance weighted such that the agent's movements are more likely to occur towards the lower end of this range, with only occasional movements reaching the maximal range, and HS weighted, such that agents favour high HS cells.

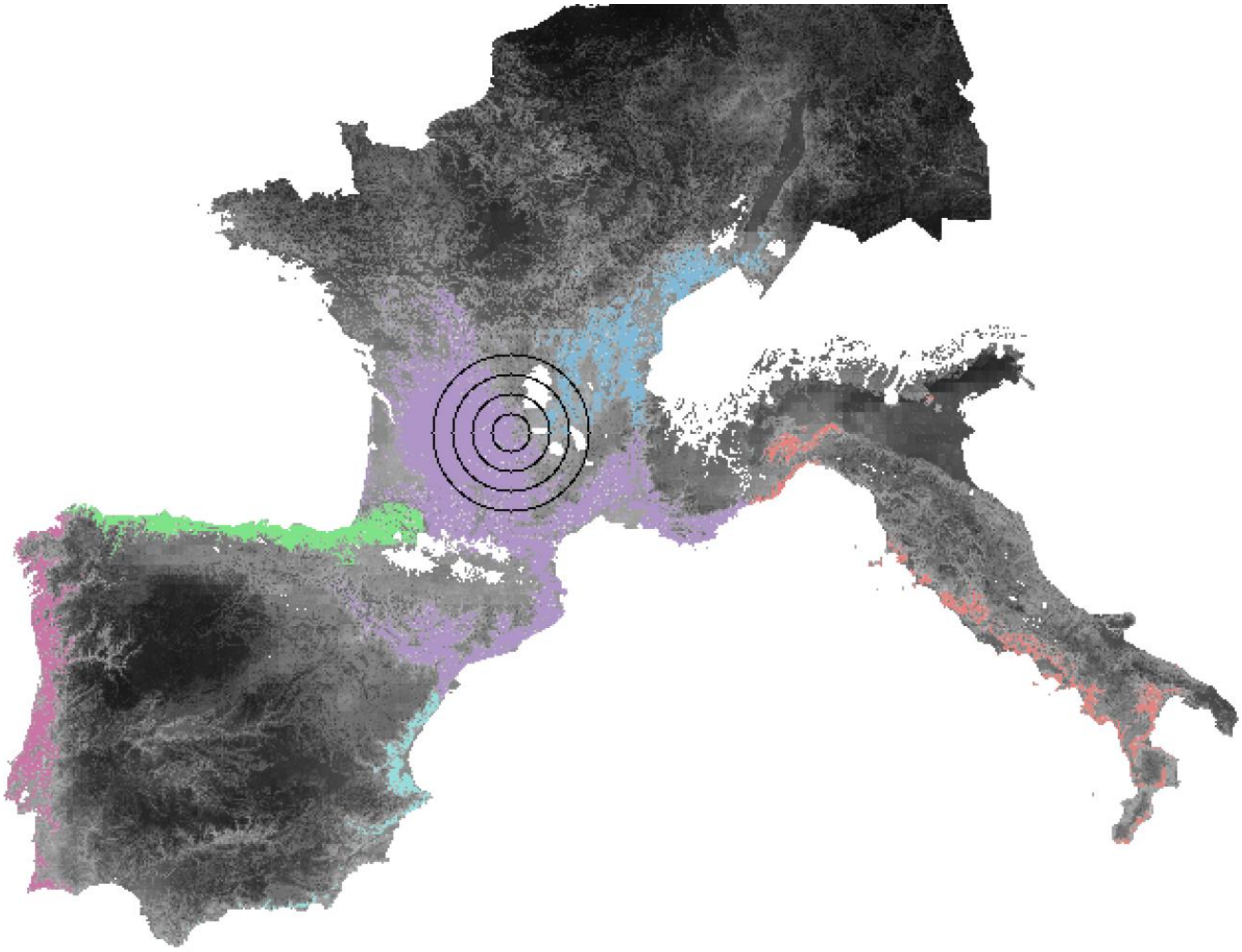

Fig. S3. An agent selected in southern France illustrates the size of the foraging radii used in Exp. 2: 50, 100, 150, and 200 km (sequential black rings). Note that with larger radii, it becomes easier for a household group to jump to other regions during foraging activities.

Experiment 2: Results

The results of experiment two show that when mortality is unaffected by the environment (HS), at the lowest foraging radius (50 km) regional populations retain a distinct genetic signature and population sizes are consistent with the size of the region. As foraging radii increase, the regional population of France increases as more neighbouring agents are able to move to the high-HS cells there (Fig. S4). High mobility (> 50km) results in inter-regional genetic exchanges creating a homogeneous genome across all regions. The increase in the proportion of France traits in the genome of the metapopulation reflects the higher initial population of that region and it’s centrality and connectedness to other regions.

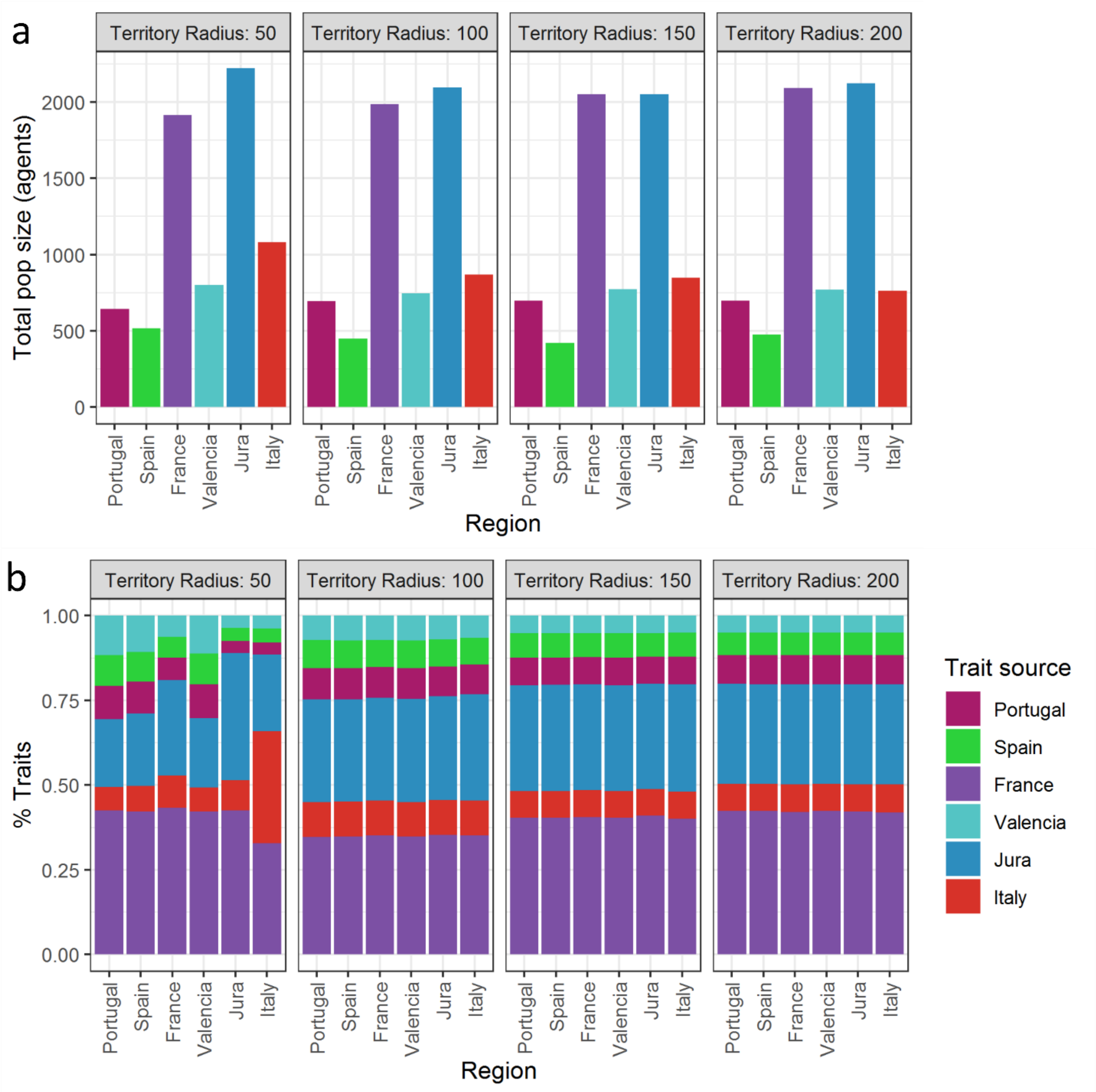

Fig. S4. Varying territory radius beyond 50 km subtly increases the proportion of the population in the Central region in southern France. This is reflected in a) the distribution of the agents across the regions and in b) the genetic make-up of the agents showing a slightly higher percentage of France traits across the regions.

With experiments 1 & 2, we have now established how the model responds to: 1) distance and HS-weighted mobility, and 2) different foraging radii. These experiments describe a *null* experimental context where habitat suitability does not affect survival. We may now investigate the impact of environmental conditions (HS) on population structure when HS affects the probability of survival (see main text).

Part 2: Model description

This supplement section is a description of our model (lgm_ecodynamics1.1) following the Overview Design and Details (ODD) Protocol described by Grimm et al. [3,4].

The model is adapted from a model by Barton and Riel-Salvatore [5] of modern human and Neanderthal interaction in Western Europe.

Overview

Purpose

lgm_ecodynamics1.0 is a modification of hominin_ecodynamics2.0 designed by Barton and Riel-Salvatore [5]. The model uses the NetLogo toolkit for coding [6]. The purpose of the current version is to experiment with the effect of behaviours governing cultural interactions and inter-regional mobility in Western Europe during the Last Glacial Maximum (LGM). The principal question we are exploring is the impact of environmental condition and different scales of mobility on the size and connectivity of regional populations, as well as their genetic makeup.

There are three primary differences between Barton and Riel-Salvatore's model and lgm_ecodynamics1.0. Firstly, we are modelling only modern humans instead of modern humans and Neanderthals so all of our agents have the same behavioural characteristics. Secondly, we use a set of six geographic markers to track inter-regional mobility rather than tracking modern human and Neanderthal genetic alleles. Finally, rather than model a homogenous landscape, we import a heterogeneous landscape based on a predictive model of habitat suitability from Burke et al. [7]. This creates discontinuities in mobility patterns in response to the availability of suitable habitat.

lgm_ecodynamics1.0 is designed to study population distribution and inter-regional gene flow under different conditions, varying the agent's foraging radius, and the impact of HS on the probability of survival. Ultimately, the model allows us to explore how the spatial distribution of suitable habitat affects the structure of the metapopulation.

Entities, state variables, and scales

Each **agent** represents a "household", i.e., a small family group of hunter-gatherers. Agents have an X,Y coordinate representing the center of their foraging territory (*home_patch*). We assign a *trait_list* to each agent that is a 2 x 100 array representing their genome. Each row of the array is a gene locus with two positions and one of six geographic alleles at each position. The agents then have a number of counter variables to track the frequency of each geographic allele within their genome. Lastly, agents have an age variable that tracks how many time-steps of the model have elapsed since they formed.

The model landscape is a raster grid of 5 x 5 km cells called **patches**. Patches are assigned a *suitability* value ranging from 0 to 1 using an imported raster map, and are based on a predictive model published elsewhere [7]. Suitability is imported as a raster map with 1 km resolution but is re-sampled on import to ~5 km resolution. Patches representing ocean or a glacier are given a null value ("NaN" or not a number in NetLogo terminology). Patches are assigned to a *core* numbered from 1 to 6 that represents a cluster of high habitat suitability (HS) patches identified during spatial analysis (see main text for GIS methodology). The majority of patches are not within one of these high HS patches and are assigned a null *core area* value. We then assign a broader *region* variable that follows the same numbering as *core area* but is not restricted to high HG clusters in a third raster. We track the cumulative land use of agents using an occupation frequency counter on each patch, *occfreq*. The *occfreq* map may be exported at the end of a run to see where on the landscape the population of agents spent the most time. Patches have a *fallow* flag that is marked *true* for one timestep following an occupation. Fallow patches are considered ineligible for movement, but are reset to *false* before the next day's movement.

Several **global** variables are assigned either by the user in the GUI or within the model setup (Table S1). These make up the behavioural parameters related to demography and mobility of the agents.

Table S1. Global model variables.

Global vars.	Description
Fradius	Foraging radius. The distance in patches an agent may move from its home_patch.
Birthrate	The probability that an agent will reproduce each timestep if in range of another agent.
trait_list_size	An optional parameter to change the length of the genome (set to 100 in the current paper).
Number	Initial population size in number of agents.
idw-weight	Parameter controlling the probability of movement at high distances from the home_patch. Higher weighting results in agents being more likely to select a closer patch. (default = 2.0)
weight-move?	Switch off for random mobility, on for HS to be factored into the probability of selecting a patch.
hs_death?	Switch on for HS to be factored into the probability of death.
hs_benefit	Percent increased survival odds on patches with suitability = 1.0 compared to the baseline at patches with suitability = 0.5. Range 0 to 1 at 0.25 increments.

Process overview and scheduling

In each time step, which represents one month, each agent moves from its *home_patch* to a patch within its foraging radius. The probability that any given patch (referred to as patches in NetLogo) is chosen is determined by its habitat suitability value, if *weight-move?* is on, and its distance from the *home_patch*, if *idw-weight* is greater than zero (also known as a roulette wheel

selection [8]). To save computational processing time, since at large foraging radii runs can take longer than a week, we use a random sub-sample of 200 patches in the weighted random selection. If any agent finds fewer than 200 patches in its radius (e.g. because many are ocean) the model switches to evaluating all patches in the radius. If *weight-move?* is off, then weighted random patch based only on distance within the foraging radius is chosen. See submodels section below for additional details on IDW and the weighted random selection of patches.

The agent then has a probability of creating a new agent at a probability equal to the birthrate. If a reproduction event is to occur, the agent looks for the nearest agent to be the "mate" and an independent assortment routine (*Indep_Assort*) routine is run to produce a combination of their *trait_lists* (see Submodels).

Lastly, we run a "death" routine which gives each agent (excepting those created during the same timestep) a probability of disbanding. The base *deathrate* is equal to the *birthrate*, but if *hs_death?* is on, that *deathrate* is adjusted based on the habitat suitability value of their patch such that lower HS decreases their probability of survival (see *hs_death* submodule below).

When the run is finished (model default is 3000 time steps representing 250 years), the model exports a wide variety of data. The most important for the present paper are: 1) the population size, or count of agents, of each *region_count* area, 2) the frequency of each genetic trait within each *region_count* area, and 3) a map of the occupation frequency of each patch (*occfreq*).

Design Concepts

Basic principles

A basic principle of this version of the model is that agents, called "groups" in the model code, maintain home range fidelity. That is, while groups are mobile within a foraging radius of their home patch, they do not venture beyond that radius. Offspring groups establish their own home within *fradius* of the location of their parent group at the time of their reproduction (which may be beyond the *fradius* of the parent group's *home_patch*).

Another basic principle is that groups are aware of and are able to predict the *suitability* of the landscape for foraging and that they use that information in mobility decisions. We referred to this capacity as spatial foresight in a previous publication [1,2]. The weighted random walk procedure estimates this behaviour without the fixed percent error used in our previous article. Agents also cannot occupy an already occupied patch or one that an agent occupied in the previous timestep, i.e. marked "fallow".

Emergence

The genetic variation within each regional population , i.e., the amount of gene flow from other regions, emerges from a combination of the group behavioural profiles (foraging radius,

birthrates, deathrates adjusted or not by habitat *suitability*), the chance pattern of interaction in a given run (i.e. path dependency) and the heterogeneous spatial distribution of habitat suitability (when *weight-move?* and/or *hs_death?* are on).

Adaptation

The environment does not change (ie. agents do not consume the habitat during foraging) and the groups do not change their behavioural profile in response to their environment. The “genetic” markers are neutral with respect to the environment, so there are no evolutionary processes over generations that affect their behaviour.

Objectives

The groups have the goal of selecting higher suitability habitat patches but they are not perfect maximisers. Rather their decision is only weighted towards patches with higher *suitability*. That is, on any given time step they **may** move from a higher suitability patch to a lower one by chance.

Learning

The groups do not learn. While they may acquire new regional traits, these traits are neutral with respect to their behaviours.

Prediction

The groups do not predict the future state of the environment (which does not change) or any aspect of other groups’ behaviour. They only read the current state of the landscape and the positions of other agents. However, it is worth remembering that the habitat suitability index incorporates several variables that encapsulate the predictability of climate conditions at a given location.

Sensing

The groups sense ocean and glacier patches and avoid them. They sense other groups within their foraging radius as part of the reproduction procedure and during movement (they avoid moving to an already occupied patch). They sense the habitat suitability values and distances of patches within their mobility radius.

Interaction

Groups affect each other’s mobility since groups may only move to empty patches. Groups interact during the *reproduction* procedure where they combine their *trait_lists* to make the offspring group’s *trait_list* (see submodels below).

Stochasticity

Several elements of the model are stochastic. By NetLogo's default, the model randomises the order in which groups take their turn within a time step. Groups' initial locations are chosen randomly from patches with a *region* number (i.e. not ocean or glaciers). For each group, movement is to a weighted randomly selected patch within the foraging radius of their *home_patch*. Probabilities of reproduction and disbanding also rely on comparing a randomly generated number to the *birthrate* and *deathrate* for each agent each time step to determine whether or not a fission or a disbanding will take place. Finally, the *Indep_Assort* procedure randomizes from which parent each allele is copied into new agent's genomes.

Collectives

Ad hoc collectives of groups determined by the agents' *region* are used for sub-setting the data analysis at the end of runs. Agents have no awareness of other agents' genomes, e.g. for use in extra attraction or avoidance behaviours.

Observation

We count the final population size of each *region*. We measure the mean allele frequencies of each region. Finally, we export a raster map showing the summed occupation frequency of each patch over the whole run (*occfreq*).

Details

Initialization

The model initializes when the user presses the setup button on the GUI. Firstly, the model imports the three raster maps, *suitability*, *core*, and *region* to create the patch landscape. It then creates a sub-set of *land* patches and scales their colour value using the *suitability* patch variable.

Next, the model creates the initial population of agents and randomly distributes them across patches with a *region* number. The agents set their *home_patch* to their current patch.

During initialization, if *hs_death?* is on, the setup also solves for *death_slope* and *death_yintercept* of a simple linear equation that will determine the variable *deathrate* on patches of different habitat *suitability*. The model assumes deathrate at suitability = 0.5 will be equal to birthrate then sets deathrate at suitability = 1.0 to be *hs_benefit* % lower. That is:

$$deathrate_{hs_{1.0}} = deathrate_{hs_{0.5}} - (birthrate * hs\_benefit)$$

The slope and y-intercept of the linear equation is solved with basic algebra:

$$death\_slope = \frac{\left(deathrate_{hs_{1.0}} - deathrate_{hs_{0.5}}\right)}{1 - 0.5}$$

$$death\_yintercept = deathrate_{hs_{1.0}} + (-1 * death\_slope)$$

Input data

No additional input data are needed other than the three raster maps.

Submodels

Reproduce: If a random floating point number between 0 and 1 falls below the *birthrate* value, the group creates an offspring and searches for the nearest other agent within its *fradius* to be its mate. If no mate is found, the offspring is simply a clone of the parent. If a mate is found then *Indep_Assort* is run to produce a combination of genomes in the offspring.

The offspring group then moves to a random unoccupied and *fallow = false* patch within *fradius* distance of the parent group's current patch, and then sets this new patch as its *home_patch*. The new group also sets its *age* to 0 so that it may skip the death routine for that time step.

Indep_Assort [mate]: This submodel is run by groups within *Reproduce* submodel and takes a target group as a parameter. For each position in the group's 2 x 100 array trait_list, an allele from a random one of the parents (the original group or the one selected to be mate) is copied into that position in the *trait_list*. The result is a randomized combination of traits that is, only on average, a 50% representation of the traits of each parent group.

hs_check_death: This submodel uses the *deathrate* parameter value to determine the likelihood of a group disbanding on a given time step. However, unlike *Reproduce*, when *hs_death?* is on it also adjusts that likelihood in response to the HS value of the group's current patch. As noted above, the base deathrate value is set equal to the birthrate during the setup procedure. In this submodel, a random floating point value between 0 and 1 is less than the result of the following formula to determine if the group disbands:

$$(death\_slope \ * suitability) \ + death\_yintercept$$

This equation linearly adjusts the *deathrate* of each group, on a per timestep basis, such that groups on higher *suitability* patches are less likely to disband. The *hs_benefit* parameter controls the magnitude of the benefit via the calculation of *death_slope* and *death_yintercept* in the model initialization (see Fig. 3).

randidw [fradius]: Random walk weighted with inverse distance weighting uses the *idw-weight* parameter to control the degree to which closer patches are favoured using the equation:

$$(d + 1)^{-w}$$

where d is distance of the patch and w is the *idw-weight* parameter. Since the circumference of a circle increases with distance, there are more patches at each sequential distance band. With an *idw-weight* of 0, or without using any inverse-distance calculation, groups are more likely to have moved farther away (Fig. S5). This is an unintuitive result of using a random walk based on random selection of patches in a specified radius. For an *idw-weight* of 1, groups are more or less equally likely to choose a patch at any distance. For an *idw-weight* of 2, groups are more likely to choose a patch that is closer. We select *idw-weight = 2.0* as our default value since shorter moves are less energetically costly and should be favoured by groups. Once the weightings are determined, the model selects one patch using the Rnd extension by Payette [8].

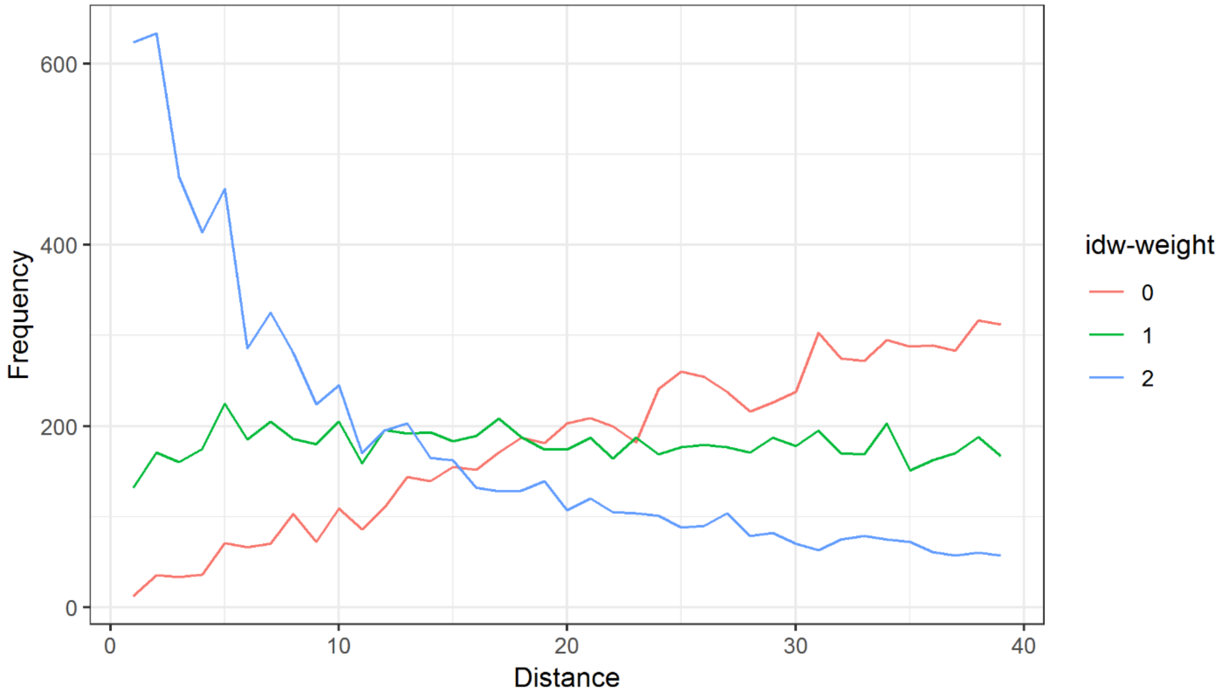

Fig. S5. Illustration of the effect of different inverse distance weights (*idw-weight*) by showing the frequency of agents' last step distances.

wrsidw [fradius]: Random walk weighted by the *suitability* patch value (*hs*) of a group's current location and inverse distance weighting:

$$hs * (d + 1)^{-w}$$

wrsidw_n200 [fradius]: Same equation as *wrsidw* but the model randomly selects 200 patches within the *fradius* for evaluation.

wrs and wrs_n200 [fradius]: If *idw-weight = 0*, the model only uses the *suitability* value in the weighted random walk. n.b. In this paper, *idw-weight* is held constant at 2 so these submodels are unused.

Part 3: GIS Regions & Core area creation

This section describes the step-by-step GIS methodology, undertaken in ArcMap 10.5, to generate the two of the input maps for the model, core and region. Rather than show the entire study area, we illustrate this process with a close-up area around what becomes a border area of our France, Jura, and Italy regions (modern Marseille, France would be roughly centered along the southern coast of this area). Fig. S5a, shows the initial habitat suitability map, coded as values from 0 to 1 that was previously published in Burke et al. [7]. Here we simply take this data as an input and do not discuss the methodology behind that landscape in this supplement.

To detect areas of clustered high habitat suitability cells, or hot-spots, we use the Hot Spot Analysis Getis-Ord Gi* tool [9,10]. This tool actually takes a vector point file as input, so we use the original grid of point data from Burke et al. [7] as the input file and its habitat suitability value as the input field. We use INVERSE_DISTANCE as the spatial relationship and EUCLIDEAN_DISTANCE and the distance method. We leave the Threshold Distance blank, which results in the tool auto-calculating a Euclidean distance such that all features have neighbors. This outputs a vector file representing a grid of points with attributes representing their classification into statistically significant bins corresponding to 90%, 95%, and 99% confidence levels. We rasterize the cells clustered above 90% confidence level (+1 to +3) into the map shown in Fig. S5b and then merge these together into a binary null or 1 in Fig. S5c.

Next, we use ArcMap's Region Group tool [10], with our rasterized binary hotspots layer as the input. We use EIGHT neighbors and WITHIN Zone Grouping to generate "groups" of contiguous cells with each group denoted with a unique sequential number, to find our main core areas (Fig. S5d). Many of these groups are very large, like our France core area, but others consist of only a handful of cells in much smaller groupings or are represented by a series of medium sized cell groups within the same general area (like our Italy core area). To reduce the number of groups and avoid dropping the small and medium groupings, we digitized large polygons be looking for pinch points between the larger groupings. In Fig. S5e, you can see these hand digitized polygons forming borders between our France and Jura core areas and between the France and Italy core areas. We then merge all smaller and medium groupings of cells with the larger groupings by using the large polygons to assign the final six core areas numbers used within the model to assign a regionally specific "genome" to our agents (Fig. S5f). The model uses the larger polygons to quantify the population size and genetic make-up of the agents at the end of each simulation run (Fig. S5e) as many agents will not be sitting within a designated core area cell as the run ends. Finally, the regions, core areas, and habitat suitability landscape are exported as ASCII rasters at 5km spatial resolution for input into the agent-based model.

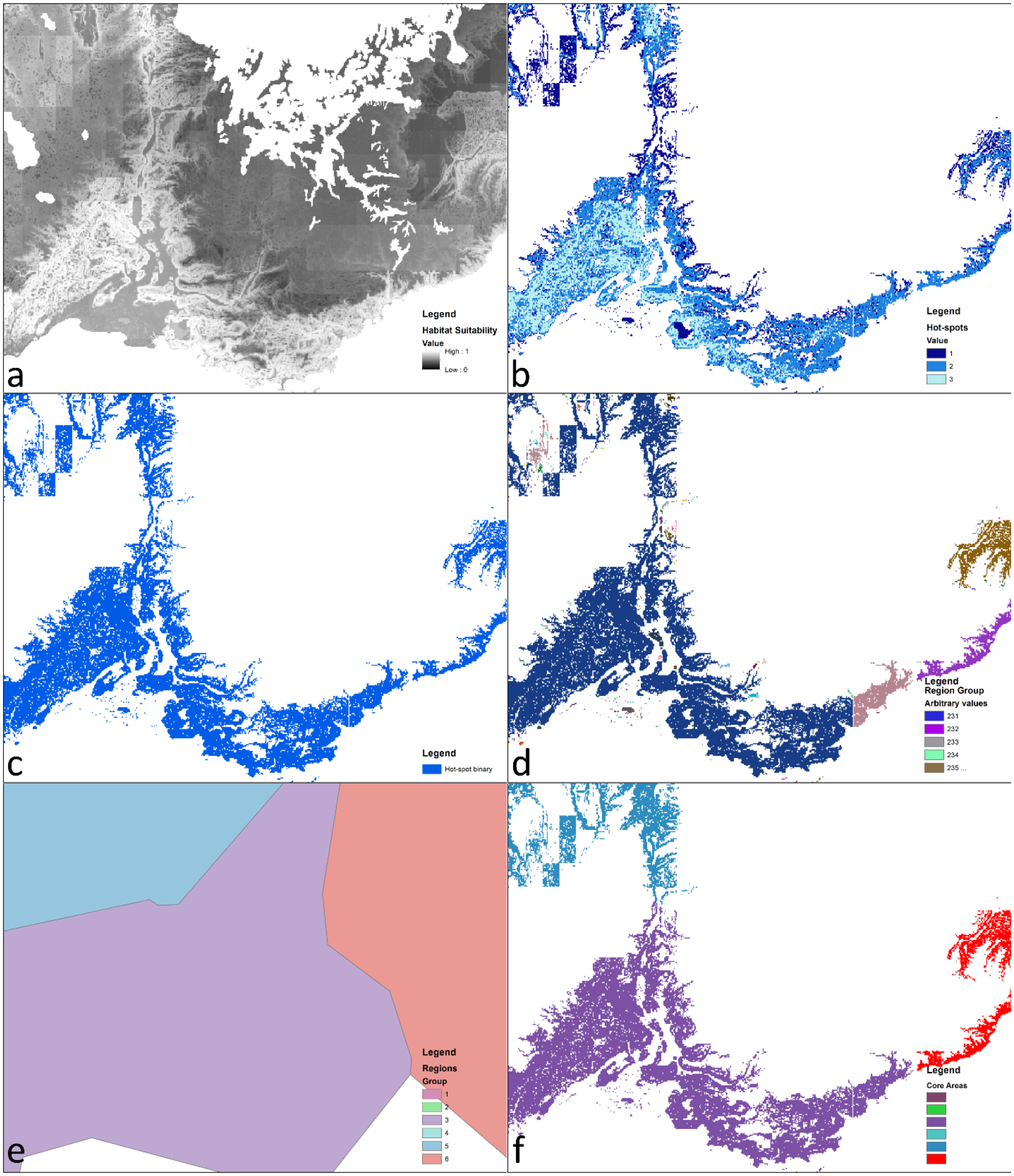

Fig. S6. Recreation of the GIS workflow including: a) input habitat suitability, b) Getis-Ord hotspots, c) reclassified binary hotspots, d) grouping of contiguous cells, e) digitized polygon regions dividing cell groups, and f) final core area divisions.

References cited

1. Wren CD, Xue JZ, Costopoulos A, Burke A. The role of spatial foresight in models of hominin dispersal. J Hum Evol. 2014;69: 70–78. doi:10.1016/j.jhevol.2014.02.004

2. Wren CD, Costopoulos A. Does Environmental Knowledge Inhibit Hominin Dispersal? Hum Biol. 2015;87: 205–223. doi:10.13110/humanbiology.87.3.0205

3. Grimm V, Berger U, Bastiansen F, Eliassen S, Ginot V, Giske J, et al. A standard protocol for describing individual-based and agent-based models. Ecol Model. 2006;198: 115–126. doi:10.1016/j.ecolmodel.2006.04.023

4. Grimm V, Berger U, DeAngelis DL, Polhill JG, Giske J, Railsback SF. The ODD protocol: A review and first update. Ecol Model. 2010;221: 2760–2768. doi:10.1016/j.ecolmodel.2010.08.019

5. Barton CM, Riel-Salvatore J. Agents of change: modeling biocultural evolution in upper pleistocene western eurasia. Adv Complex Syst. 2012;15: 1150003. doi:10.1142/S0219525911003359

6. Wilensky U. NetLogo [Internet]. Evanston: Center for Connected Learning and Computer-Based Modeling, Northwestern University; 1999. Available: http://ccl.northwestern.edu/netlogo

7. Burke A, Kageyama M, Latombe G, Fasel M, Vrac M, Ramstein G, et al. Risky business: The impact of climate and climate variability on human population dynamics in Western Europe during the Last Glacial Maximum. Quat Sci Rev. 2017;164: 217–229. doi:10.1016/j.quascirev.2017.04.001

8. Payette N. NetLogo Rnd-Extension [Internet]. 2013. Available: https://github.com/NetLogo/Rnd-Extension

9. Ord JK, Getis A. Local Spatial Autocorrelation Statistics: Distributional Issues and an Application. Geogr Anal. 1995;27: 286–306. doi:10.1111/j.1538-4632.1995.tb00912.x

10. ESRI. ArcGIS [Internet]. Redlands, California: Environmental Systems Research Institute; 2016. Available: http://desktop.arcgis.com/en/arcmap/10.5/
